# CAG Repeat Not Polyglutamine Length Determines Timing of Huntington’s Disease Onset

**DOI:** 10.1016/j.cell.2019.06.036

**Published:** 2019-08-08

**Authors:** Genetic Modifiers of Huntington’s Disease (GeM-HD) Consortium, Jong-Min Lee, Kevin Correia, Jacob Loupe, Kyung-Hee Kim, Douglas Barker, Eun Pyo Hong, Michael J. Chao, Jeffrey D. Long, Diane Lucente, Jean Paul G. Vonsattel, Ricardo Mouro Pinto, Kawther Abu Elneel, Eliana Marisa Ramos, Jayalakshmi Srinidhi Mysore, Tammy Gillis, Vanessa C. Wheeler, Marcy E. MacDonald, James F. Gusella, Branduff McAllister, Thomas Massey, Christopher Medway, Timothy C. Stone, Lynsey Hall, Lesley Jones, Peter Holmans, Seung Kwak, Anka G. Ehrhardt, Cristina Sampaio, Marc Ciosi, Alastair Maxwell, Afroditi Chatzi, Darren G. Monckton, Michael Orth, G. Bernhard Landwehrmeyer, Jane S. Paulsen, E. Ray Dorsey, Ira Shoulson, Richard H. Myers

**Keywords:** Huntington’s disease, CAG repeat, trinucleotide repeat, genetic modifier, age at onset, somatic DNA expansion, DNA maintenance, DNA repair, polyglutamine disease, disease modification

## Abstract

Variable, glutamine-encoding, CAA interruptions indicate that a property of the uninterrupted *HTT* CAG repeat sequence, distinct from the length of huntingtin’s polyglutamine segment, dictates the rate at which Huntington’s disease (HD) develops. The timing of onset shows no significant association with *HTT cis*-eQTLs but is influenced, sometimes in a sex-specific manner, by polymorphic variation at multiple DNA maintenance genes, suggesting that the special onset-determining property of the uninterrupted CAG repeat is a propensity for length instability that leads to its somatic expansion. Additional naturally occurring genetic modifier loci, defined by GWAS, may influence HD pathogenesis through other mechanisms. These findings have profound implications for the pathogenesis of HD and other repeat diseases and question the fundamental premise that polyglutamine length determines the rate of pathogenesis in the “polyglutamine disorders.”

## Highlights

• Uninterrupted CAG repeat, not polyglutamine, size drives the timing of HD onset
• HD age at onset is influenced by at least six genes involved in DNA maintenance
• Genetic modifier loci often show both onset-delaying and onset-hastening haplotypes
• The rate-determining mechanism is likely to be somatic expansion of the CAG repeat

## Graphical Abstract

**Figure:**
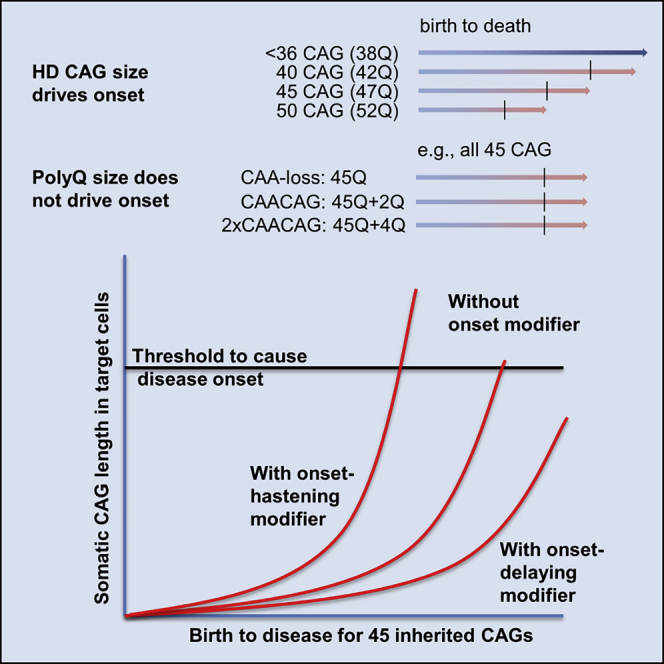

## Teaser

The onset of Huntington’s disease is shown to be dependent on the size of the uninterrupted CAG repeat sequence, not polyglutamine.

## Introduction

Huntington’s disease (HD) is the most frequent of the polyglutamine diseases: dominantly inherited neurological disorders in which an expanded CAG trinucleotide repeat lengthens a segment of encoded glutamines in a particular protein (Lieberman et al., 2018). In all such diseases, age at onset is inversely correlated with mutant CAG repeat length (Gusella and MacDonald, 2000), but each presents distinct neuropathology and clinical manifestations. These distinctive features are usually assumed to result from a length-dependent effect of polyglutamine on the expression, activity, and/or functional interactions of the encoded protein or of its polyglutamine-containing fragments, which show a propensity for aggregation. In HD, the expanded CAG tract is in exon 1 of *HTT*, which encodes huntingtin, a large (>340 kDa), largely α-helical HEAT (huntingtin, elongation factor 3, protein phosphatase 2A, and lipid kinase TOR) repeat protein (Guo et al., 2018). The mutation results in progressive neuronal loss most prominently in the striatum and other basal ganglia structures but also throughout the cerebral cortex. Subtle alterations in brain can be detected a decade or more before clinical onset (Paulsen et al., 2008). Clinically, HD is diagnosed by its motor effects, but the disease also affects cognition and behavior and leads to death ∼15 years after motor onset (Keum et al., 2016). Although expression of long polyglutamine tracts in huntingtin or huntingtin fragments can cause a variety of subtle to extreme phenotypic consequences in cultured cells or model organisms (ranging from yeast to large mammals), the relevance of these phenotypes to the human disease remains uncertain and they have not led to disease-modifying treatments. Current hopes for an effective intervention lie in suppressing expression of mutant huntingtin (Wild and Tabrizi, 2017), but additional therapeutic approaches are needed to prevent or delay onset or progression of this devastating disorder.

While expanded *HTT* CAG repeat size explains ∼60% of the individual variation in HD age at onset, the remaining variation shows heritability (Djoussé et al., 2003). This prompted a human genetics strategy to identify disease-modifying factors that act prior to clinical diagnosis to either delay or hasten onset, with the expectation that, since they are validated in humans, these disease-modifying genes could reveal biochemical processes to target in drug development (Genetic Modifiers of Huntington’s Disease (GeM-HD) Consortium, 2015). We previously carried out a genome-wide association study (GWAS) of 4,082 HD individuals using the difference between age at onset predicted by CAG length and actual age at onset of motor symptoms. This GWAS found three significant modifier signals at two loci, with pathway analysis suggesting DNA maintenance and mitochondrial regulation as potential modifying processes. We have now extended this GWAS strategy to over 9,000 HD individuals. The increased power has detected both infrequent modifier alleles of strong effect and common modifiers with more modest impact at new loci and separately has highlighted rare subjects for whom the standard *HTT* PCR fragment-based genotyping assay mis-estimated CAG repeat length due to variations in glutamine-encoding CAA interruptions. These individuals distinguish the effect of CAG repeat length and polyglutamine length and reveal that the rate-determining driver (“rate driver”) dictating the timing of motor onset is not the length of polyglutamine in huntingtin, but the length of the uninterrupted CAG repeat in *HTT*. Although these findings indicate that the timing of onset is determined by a property of the expanded CAG repeat rather than the length of polyglutamine that it encodes, they do not reveal what drives the resultant toxicity (“toxicity driver”) and do not exclude an effect of polyglutamine from that later role. Overall, the GWAS results reveal several additional loci and modifier effects that cement a role for DNA maintenance mechanisms in modifying the timing of disease onset and disclose new modifier loci that may act through other mechanisms. The DNA maintenance modifier genes most likely influence somatic expansion of the *HTT* CAG repeat suggesting this process as a potential therapeutic target to delay or prevent HD onset.

## Results

### Genome-wide Association Analysis

**Figure S1:**
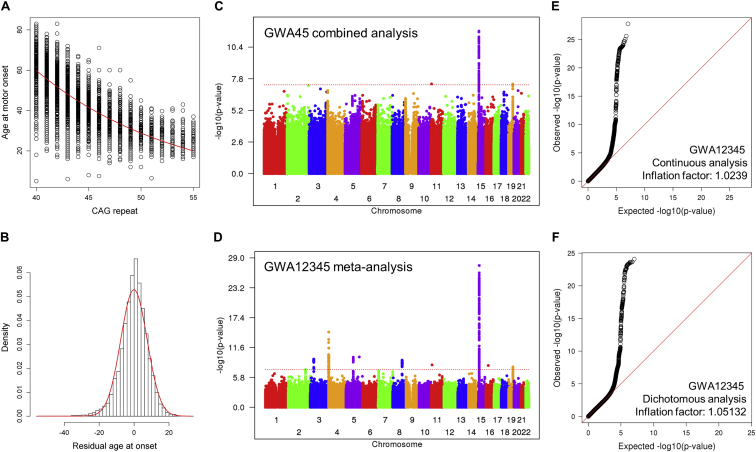
Residual Age at Onset Phenotype for GWA Analysis to Identify Genetic Modifiers of HD, Related to Figure 1 **(**A) Age at onset data for individuals with HD (y axis) were compared to CAG repeat size based on genotyping. A red line represents our standard CAG-onset phenotype model. Residual age at onset was calculated for each subject by subtracting expected age at onset based on our CAG-onset model from observed age at motor onset. (B) Residual age at onset was our primary phenotype for genetic analysis. Distribution of residual age at onset of individuals with HD (histogram) was compared to a theoretical normal distribution based on the mean and standard deviation of actual data to confirm data normality. (C) For an independent comparison with our previous GWA results (GWA123), we performed mixed effect model GWA analysis of additional HD individuals (GWA45). The Manhattan plot summarizes association signals using residual age at onset for 4,793 HD individuals. Y- and x axis represent -log10(p value) and chromosome number. (D) In addition, we performed and subsequently compared 1) meta-analysis to summarize 5 independent GWA analysis results and 2) mixed effect model combined analysis to avoid statistical artifacts. The Manhattan plot of the meta-analysis shown here and that of combined analysis using the continuous phenotype in Figure 1 were very similar, confirming the lack of batch effect in our GWA analysis results. In order to provide meaningful effect sizes of associated loci, we primarily used the results of the combined analysis. (E) A quantile-quantile (QQ) plot based on the GWA analysis results using continuous phenotype (mixed effect model, combined analysis) and the inflation factor confirmed the lack of statistical inflation in our results. (F) A QQ plot based on the GWA analysis results using dichotomous phenotype (fixed effect model, combined analysis) and the inflation factor confirmed the lack of statistical inflation in our results.

**Figure 1:**
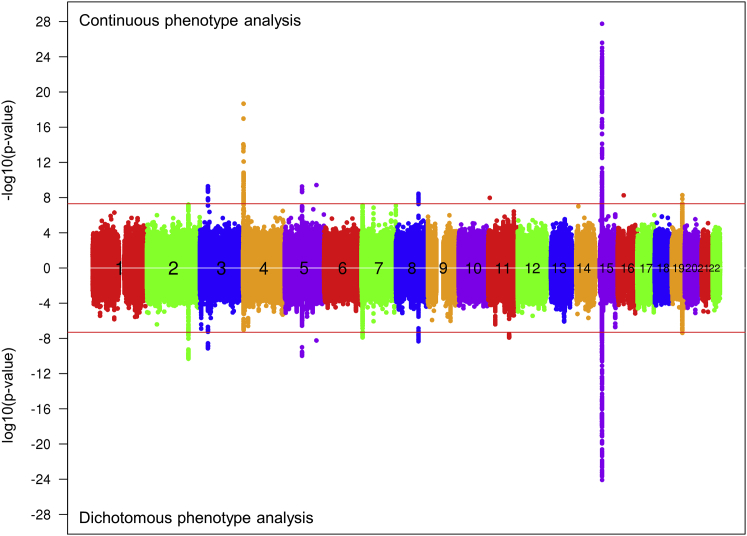
Age at Onset GWAS Signals GWAS results using the residual age at onset phenotype or a dichotomized phenotype, with each circle representing a test SNP and significances shown as –log10(p value) (top) and log10(p value) (bottom), respectively. See also Figures S1, S2, and S3 and Tables S2 and S3–S5.

Our previous “GWA123” to identify genetic factors that influence age at onset used DNA from HD individuals with 40–55 CAG repeats genotyped in three stages and analyzed using a robust statistical phenotype model relating CAG repeat length to log-transformed age at onset of diagnostic motor signs (Genetic Modifiers of Huntington’s Disease (GeM-HD) Consortium, 2015). This yielded a residual age at onset value for each subject that was transformed back into natural scale as a phenotype for continuous, quantitative association analysis. Here, additional European-ancestry HD individuals from Registry (Orth et al., 2010) and the Enroll-HD platform (Landwehrmeyer et al., 2016) were genotyped in separate waves (Figure S1A). The distribution of residuals for the combined GWA12345 dataset was similar to a theoretical normal distribution (Figure S1B). We re-imputed each GWA dataset using the Haplotype Reference Consortium (HRC; McCarthy et al., 2016) and then analyzed the 9,064 unique individuals (4,417 males; 4,647 females) in the combined GWA12345 dataset using two parallel approaches: continuous analysis of association to residual age at onset and dichotomous analysis of extremes of residual age at onset (Figure 1). The former strategy is more effective for detecting rare modifier alleles, whereas the latter is less influenced by slight imprecision in establishing age at onset since it compares allele frequencies between groups with onset substantially later or earlier than expected. Results from the combined GWA45 dataset alone and from all GWA datasets subjected to meta-analysis are shown in Figures S1C and S1D along with Q-Q plots for the GWA12345 continuous and dichotomous analyses in Figures S1E and S1F.

### An Unexpected Signal near *HTT*

**Figure S2:**
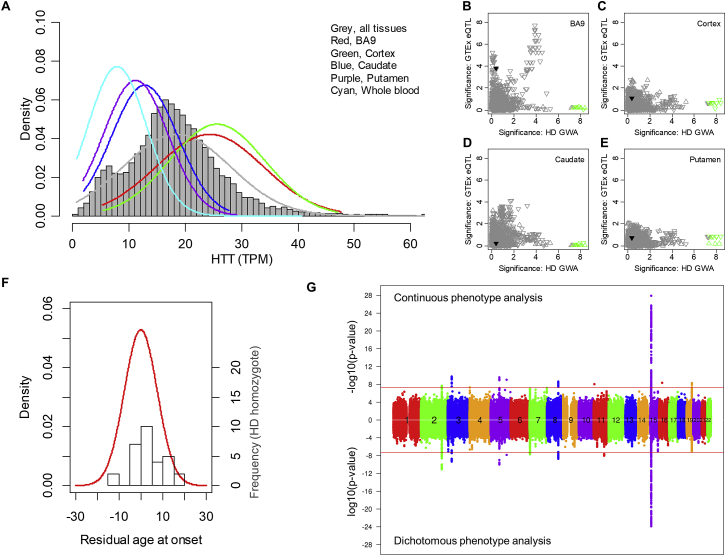
Chr 4 GWAS Signals Do Not Correspond to *HTT cis*-eQTL Signals and Are Dramatically Reduced by Correcting Mis-estimated CAG Repeat Length, Related to Figures 1 and 2 (A) Expression levels of GTEx subjects in various tissues are plotted. The background histogram represents the distribution of *HTT* mRNA levels across all tissues. TPM (x axis) represents Transcripts Per Million (Li et al., 2010). (B-E) GWAS signals for chromosome 4 (x axis) were compared to GTEx eQTL analysis results for prefrontal cortex BA9 (C), cortex (D), caudate (E), and putamen (F). Upward and downward triangles represent SNPs whose minor alleles were associated with increased and decreased *HTT* mRNA levels, respectively, in GTEx data. SNPs on the haplotype marked by rs764154313 are infrequent and therefore were filtered out from publicly available GTEx eQTL results. Some SNPs from the haplotype tagged by rs183415333 are in the GTEx eQTL dataset and are compared to HD modifier GWAS data (green triangles) but do not correspond with eQTLs in any of the brain tissues. In BA9, GWAS signals in the 1E-4 range contributed by more frequent SNPs correspond with eQTL signals for decreased *HTT* expression, but these weak GWAS signals are removed by conditioning for rs183415333. Notably, the rs183415333-tagged haplotype represents a small subset (∼10%) of the chromosomes that bear the more frequent rs13102260 promotor SNP (black triangle here and in Figure 2)A, which was proposed to be involved in regulation of *HTT* expression levels and subsequent modification of HD (Bečanović et al., 2015) but which failed to yield a strong association signal in either GWA123 or GWA12345. (F) Residual age at onset of heterozygous HD individuals (red line; primary y axis) is qualitatively compared to a histogram distribution of residual age at onset of HD individuals with 2 expanded alleles (30 individuals; secondary y axis). (G) Correcting the mis-estimated CAG repeat length of individuals with rs764154313 or rs183415333 minor alleles dramatically reduced apparent chr 4 signals without a major change in all other GWAS signals. The GWAS data from Figure 1 were re-analyzed using the true uninterrupted CAG repeat from MiSeq sequencing of rs764154313 or rs183415333 minor allele subjects. Of 29 individuals with the rs764154313 minor allele, 28 had DNA available for sequencing: 21 had a CAA-loss allele on the disease chr 4, 2 had CAA-loss allele on the normal chr 4 and 5 had a canonical sequence on both chr 4. Of 102 individuals with the rs183415333 minor allele, 98 had DNA available: 67 had a CAACAG-duplication allele on the disease chr 4, 22 had a CAACAG-duplication allele on the normal chr 4, 2 had a CAACAG-duplication on both the normal and the expanded chr 4 and 7 had a canonical sequence on both chr 4. The 5 individuals for whom no DNA sequence was available, along with one canonical allele individual whose sample showed evidence of mix-up, were excluded from this analysis of 9058 individuals. In this dataset, the length of the CAG repeat accounted for 57% of the variance in age at onset. Inclusion of all SNPs with p < 1E-5 and p < 1E-3 raised the explanatory power to 66% and 90%, respectively. However, to what degree this polygenic modification score is predictive remains to be tested in an independent HD sample. Of note, based on the ancestry of some HD individuals in our current dataset with these CAACAG-duplication alleles, this *HTT* haplotype tagged by the rs183415333 minor allele is likely to correspond to that reported based upon PCR fragment-based genotyping to be associated with later than expected onset in a Danish HD family (Nørremølle et al., 2009).

**Figure 2:**
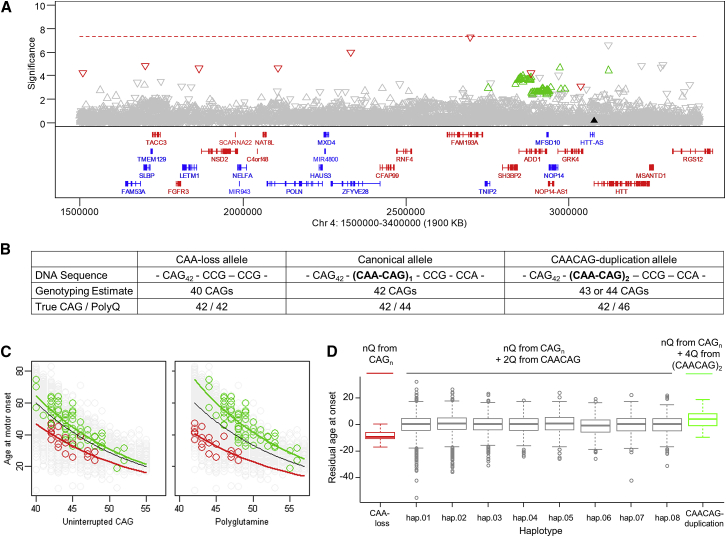
Correcting Mis-estimated CAG Repeat Length Removes Significant Signals at *HTT* (A) Chr 4 signals (–log10(p value); continuous phenotype) plotted versus genomic coordinate (GRCh37/hg19). The dotted red line indicates genome-wide significance. Red and green symbols are SNPs tagging haplotypes with minor alleles of rs764154313 and rs183415333, respectively. In this and subsequent plots, downward and upward triangles are SNPs with minor alleles associated with hastened and delayed onset, respectively. Genes are below in red (plus strand) and blue (minus strand). (B) *HTT* repeat and adjacent sequence, CAG repeat length estimated by genotyping, true CAG repeat length, and polyglutamine length for chrs with CAA-loss (left), canonical (center), and CAACAG-duplication haplotypes, using 42 uninterrupted CAGs as an example. (C) MiSeq analysis of individuals with rs764154313 and rs183415333 minor alleles identified individuals with CAA-loss (red) and CAACAG-duplication (green) alleles (all others shown in gray), permitting comparison of their age at onset with uninterrupted CAG repeat length (left) or total polyglutamine length (right). The black trend line represents our standard onset-CAG phenotyping model for comparison with trend lines in red and green for those with the CAA-loss and CAACAG-duplication alleles, respectively. (D) A boxplot (plotted as quartiles: whiskers1.5∗IQR(interquartile range)) of residual age at onset for individuals with a rare CAA-loss HD haplotype (red; N = 21), the 8 most frequent canonical (single CAACAG) HD haplotypes (gray; N = 3357 hap.01, 2016 hap.02, 942 hap.03, 272 hap.04, 266 hap.05, 312 hap.06, 257 hap.07, 302 hap.08) or a rare CAACAG-duplication HD haplotype (green; N = 69), ordered by increasing polyglutamine length (given the same CAG repeat length). See also Figure S2 and Table S1.

The most frequent 7 *HTT* SNP haplotypes, accounting for >83% of HD chromosomes (chr), are not associated with differences in age at onset (Lee et al., 2012a). Neither is age at onset influenced by the normal chr *HTT* haplotype or CAG repeat size (Lee et al., 2012b). Consequently, the striking emergence of an apparent GWAS signal on chr 4 near *HTT* was unexpected. Conditional analysis based upon the top SNP resolved this apparent hit into two independent signals, tagged by rare alleles, that seemed to be associated with earlier (rs764154313, minor allele frequency [MAF] = 0.2%, p = 2.1E-19) and later (rs183415333, MAF = 0.6%, p = 1.4E-14) than expected onset, respectively. We examined whether genetic effects on *HTT* mRNA production were responsible since an *HTT* promotor SNP, rs13102260, was recently proposed to alter HD onset through an effect on *HTT* expression on both normal and disease chrs (Bečanović et al., 2015). In normal brain data from the GTEx Consortium, *HTT* mRNA shows wide variation in expression (Figure S2A) but significant *cis*-eQTL SNPs show no correspondence with the GWAS signals (Figures S2B–S2E). Notably, rs13102260 is not significant (black triangle, Figures 2A and S2B–S2E). Also, HD individuals with two expanded CAG alleles express twice the level of mutant huntingtin but have age at onset residuals (based upon the longer expanded repeat) similar to HD heterozygotes (Figure S2F). Taken together, these findings suggest that variation in *HTT* mRNA expression within the normal physiological range does not significantly influence HD age at onset.

### HD Age at Onset Is Determined by CAG Repeat Length, Not Polyglutamine Length

We next considered the possibility of a change to the *HTT* CAG repeat sequence. The vast majority (> 95%) of European ancestry HD chrs carry a canonical CAG repeat region with an uninterrupted CAG repeat followed by CAACAG (Figure 2B; Table S1). Since CAA and CAG both encode glutamine, the polyglutamine size produced is consistently greater by 2 residues than the uninterrupted CAG repeat, so it has not been possible to distinguish whether it is the polyglutamine or CAG repeat size that determines the timing of HD onset. For GWA12345, uninterrupted CAG repeat length was estimated by a widely used PCR fragment-sizing assay, relative to sequenced canonical DNA standards. We reasoned that rare non-canonical sequence variations could result in inaccurate estimation of the uninterrupted CAG repeat. The resulting artificial effect on residual age at onset might then produce the unexpected chr 4 signals.

Indeed, sequencing of the *HTT* CAG repeat region in individuals with the rs764154313 minor allele revealed a polymorphism involving loss of the CAA interruption (Figure 2B; Gellera et al., 1996) such that the genotyping assay underestimated the length of the uninterrupted CAG repeat by 2 CAGs. Conversely, individuals with the rs183415333 minor allele showed a polymorphism comprising a second CAA interruption (Pêcheux et al., 1995), two codons upstream of the first (formally equivalent to CAACAG duplication following the uninterrupted CAG repeat). The overestimation of the uninterrupted CAG repeat length for these CAACAG-duplication chrs is 1 or 2 residues rather than always 2, due to mis-priming of the genotyping PCR primer on this sequence variation. When the true uninterrupted CAG length rather than the mis-estimated length was used, the GWAS signal was greatly reduced to the border of significance for rs764154313 (p = 5.0E-8) and below genome-wide significance for rs183415333 (p = 2.2E-5; Figure 2A), indicating that the original highly significant chr 4 GWAS signals were artifacts of mis-estimated expected age at onset due to mis-estimation of the true uninterrupted CAG repeat length.

Importantly, the CAA-loss and CAACAG-duplication alleles allowed the role of uninterrupted CAG repeat length to be distinguished from polyglutamine length in determining age at onset. The CAA-loss chrs encode the same number of glutamines as consecutive CAGs while the CAACAG-duplication chrs specify 4 more glutamines than consecutive CAGs (Figure 2B). As demonstrated in Figure 2C, regardless of haplotype or encoded polyglutamine, age at onset tracks best with uninterrupted CAG repeat length, with longer CAG alleles associated with earlier onset. Therefore, it is the length of the CAG repeat expansion mutation itself that is the rate driver for onset. If the timing of onset was determined by polyglutamine length-dependent toxicity, then, for identical uninterrupted CAG sizes, CAACAG-duplication alleles that encode 4 more glutamines than CAA-loss alleles should drive earlier onset. However, it is clear in comparison to CAA-loss alleles that neither the greater polyglutamine length encoded by CAACAG-duplication alleles (+4Q) nor CAACAG-canonical alleles (+2Q) results in earlier-than-expected onset (Figure 2D). Rather, a comparison of CAA-loss and CAACAG-duplication chrs carrying identical uninterrupted CAG repeats (choosing any individual CAG in Figure 2C) shows that age at onset is consistently later for individuals with a CAACAG-duplication allele, even though these alleles specify 4 more glutamines than a CAA-loss allele.

These findings indicate that the rate driver for the timing of HD onset lies in some property of the uninterrupted CAG repeat separate from its glutamine coding property. A similar conclusion was reported by others while this paper was in review (Wright et al., 2019). However, it is important to state that this does not preclude a role for polyglutamine in HD, since it remains a candidate for the toxicity driver that acts after the CAG repeat length has determined the timing of onset. The far weaker signals for rs764154313 and rs183415333 after correcting the CAG repeat length, if meaningful, suggest the potential for subtle functional differences attributable to non-CAG sequences on these haplotypes. The differences do not lie in polyglutamine toxicity since, as noted above, the longer polyglutamine on CAG-expanded rs183415333-tagged chrs is associated with later rather than earlier onset. The effects are not due to normal chrs with these tag SNPs, since removing only individuals with the tag SNPs on the disease chr eliminates the remaining signal. From the previous eQTL analyses, *HTT* mRNA levels are not likely to be responsible, although altered regulation under special circumstances or altered translation are formally possible. Similarly, it is conceivable that, after CAG length correction, these SNPs weakly capture a modifier influence acting through another gene distal to *HTT.* However, Occam’s razor suggests a more likely hypothesis for a potential effect of haplotype separate from the CAG repeat itself. The critical importance of a DNA sequence property as the rate driver for HD onset suggests that the DNA sequence context in which the CAG repeat is embedded may also have an influence. On CAA-loss chrs, the CAG repeat is followed by CCG_12_CCT_2_CAGCTTCCT_1_ and on CAACAG-duplication chrs, it is followed by CAA_1_CAG_1_CAA_1_CAG_1_CCG_1_CCA_1_CCG_7_CCT_3_CAGCTTCCT_1._ Sequencing of samples not tagged by rs764154313 or rs183415333 confirmed correct PCR-assay genotyping of the uninterrupted CAG sequence on canonical chrs and showed that the vast majority carry CAA_1_CAG_1_CCG_1_CCA_1_CCG_7_CCT_2_CAGCTTCCT_1_ after the CAG repeat (Table S1). An influence of the downstream sequences on the rate-determining property of the uninterrupted CAG repeat might then explain the remaining weaker GWAS signals. Similarly, other sequence variations on even less frequent HD chrs might also influence age at onset but not be detected with the current GWAS sample size. Indeed, the sequences of a selection of other HD and normal chrs revealed a wide variety of rarer non-canonical sequence variations (Table S1). DNA sequencing of a much larger HD sample will be needed to address possible effects of background haplotype and CAG repeat sequence context, if any, on disease onset and manifestations but could inform therapeutic targeting of this *HTT* region.

### DNA Maintenance Genes Modify HD Age at Onset

Correction of the mis-estimated CAG repeats did not have a major effect on the GWAS results other than to dramatically reduce the chr 4 signals (Figure S2G), so we proceeded to examine the multiple loci implicated by genome-wide significance in GWA12345 as modifiers of HD age at onset (Table 1). In GWA123, we had observed modifier loci on chr 8 and chr 15, with the latter exhibiting two independent opposing effects (Genetic Modifiers of Huntington’s Disease (GeM-HD) Consortium, 2015), and in follow-up studies a chr 3 locus achieved genome-wide significance (Lee et al., 2017). These loci emerged again in GWA12345, along with several new loci genome-wide significant in either continuous or dichotomous analysis or both. Like the chr3 (*MLH1*) and chr15 (*FAN1*) loci, new loci on chr 2 (*PMS1*), 5 (*MSH3/DHFR*), 7 (*PMS2*), and 19 (*LIG1*) all contain genes associated with DNA repair, but, like the chr 8 locus (*RRM2B/UBR5*), additional modifier sites on chr 5 (*TCERG1*) and chr 11 (*CCDC82*) may not be directly connected to such processes. Two additional loci, on chr 11 (*SYT9*) and chr 16 (*GSG1L*), displayed significant signal only from a single very low-frequency SNP allele, suggesting a statistical artifact due to extreme phenotypic outliers. A larger sample size and/or functional analysis will be needed to firmly establish these loci as *bona fide* modifiers.

**Table 1:** Genome-wide Significant Loci with Additional Modifier Haplotypes Identified by Conditional Analysis

Chr	Modifier^a^	Top SNP	BP (hg19)	Minor Allele	MAF (%)	Continuous Analysis						Dichotomous Analysis	Candidate Modifier Genes
						Overall	p Value	Males	p Value	Females	p Value	Overall	
						Effect Size (years)		Effect Size (years)		Effect Size (years)		p Value	
1	1AM1^b^	rs567500111	164283625	A	0.3	−4.4	6.9E-06	−8.0	2.0E-08	−1.3	3.5E-01	6.5E-02	
2	2AM1	rs3791767	190639915	C	20.7	−0.8	6.3E-08	−0.9	6.3E-06	−0.7	9.5E-04	4.9E-11	*PMS1*
3	3AM1	rs1799977	37053568	G	31.0	0.8	5.1E-10	0.6	1.3E-03	0.9	3.3E-08	7.3E-10	*MLH1*
	5AM1	rs701383	79913275	A	25.7	−0.8	5.5E-10	−0.4	2.1E-02	−1.2	4.6E-11	1.0E-10	
5	5AM2	rs113361582	80086504	G	0.3	6.1	1.3E-09	6.1	3.4E-05	6.4	3.1E-06	6.6E-05	*MSH3*, *DHFR*
	5AM3	rs1650742	79990883	G	33.1	0.6	1.6E-06	0.3	7.3E-02	0.9	2.2E-07	1.0E-06	
5	5BM1	rs79727797	145886836	A	2.4	2.3	3.8E-10	2.7	2.5E-07	2.0	1.1E-04	5.8E-09	*TCERG1*
7	7AM1	rs74302792	6079993	A	15.9	0.8	7.4E-08	0.9	4.1E-05	0.8	2.8E-04	1.3E-08	*PMS2*
8	8AM1	rs79136984	103213640	T	8.2	−1.2	3.6E-09	−1.4	1.9E-06	−1.0	2.3E-04	4.7E-09	*RRM2B*, *UBR5*
11	11AM1	rs7936234	96106737	A	19.6	0.6	1.7E-05	0.7	5.5E-04	0.6	5.5E-03	1.3E-08	*CCDC82*
11	11BM1^b^	rs79714630	7303052	G	0.1	−9.6	1.1E-08	−10.6	7.0E-06	−8.6	3.1E-04	3.8E-02	*SYT9*
12	12AM1^b^	rs140253376	108992727	A	0.2	−6.1	8.3E-06	−10.1	3.0E-08	−1.2	5.8E-01	6.9E-03	
	15AM1	rs150393409	31202961	A	1.4	−5.2	1.8E-28	−5.1	6.3E-14	−5.4	2.1E-16	1.6E-17	
15	15AM2	rs35811129	31241346	A	27.5	1.3	9.4E-26	1.1	1.3E-09	1.6	1.8E-19	8.2E-25	*FAN1*
	15AM3	rs151322829	31197995	T	0.7	−3.8	1.4E-08	−4.1	1.0E-05	−3.5	2.3E-04	4.3E-07	
	15AM4	rs34017474	31230611	C	38.2	0.8	8.5E-11	0.5	3.0E-03	1.0	1.1E-10	2.6E-11	
16	16AM1^b^	rs187055476	27873637	G	0.3	−6.1	5.5E-09	−7.2	7.1E-07	−5.1	8.1E-04	2.2E-04	*GSG1L*
18	18AM1^b^	rs530017366	56126806	T	0.2	−5.1	1.2E-04	−12.0	8.2E-09	−0.1	9.5E-01	4.3E-01	
	19AM1	rs274883	48622545	G	16.7	0.9	5.3E-09	0.8	7.3E-05	0.9	1.8E-05	1.1E-07	
19	19AM2	rs3730945	48645976	G	37.1	−0.6	5.8E-07	−0.5	6.8E-03	−0.7	1.2E-05	2.4E-06	*LIG1*
	19AM3	rs145821638	48620943	A	0.1	7.7	1.5E-06	3.8	1.3E-01	10.2	1.1E-06	7.5E-03	

**Figure S3:**
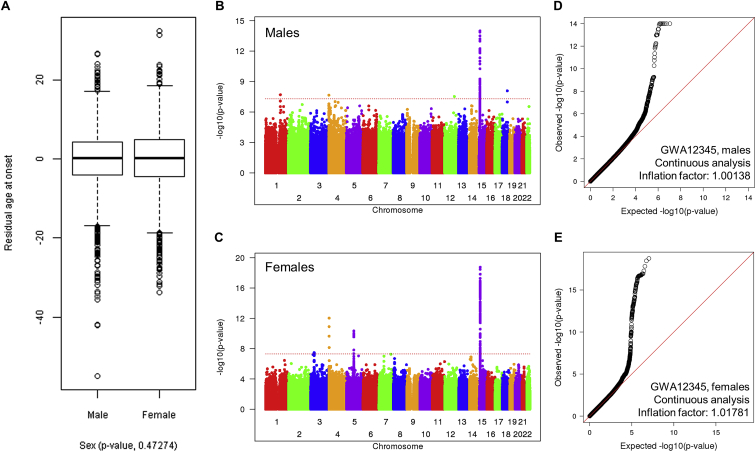
Sex-Specific Association Analysis of Residual Age at Onset, Related to Figure 1 and Table 1 (A) The distribution of age at onset residuals in GWA12345 subjects is presented as a standard boxplot by sex, showing no significant difference between males and females. (B) The Manhattan plot summarizes association signals using residual age at onset for 4,417 male HD individuals. Y- and x axis represent -log10(p value) and chromosome number. (C). The Manhattan plot summarizes association signals using residual age at onset for 4,647 female HD individuals. (D) A QQ plot based on the male-specific GWA analysis is shown, confirming lack of statistical inflation. (E) A QQ plot based on the female-specific GWA analysis is shown, confirming lack of statistical inflation.

Ref, reference; Alt, Alternate, MAF, minor allele frequency. See also Figure S3 and Tables S2 and S3–S5.

a For clarity of presentation, to avoid confusion with haplotypes defined by different means, and in anticipation of discovering additional modifier loci on some of these chrs, we have adopted a naming system for the haplotypes marking the modifier effects that indicates the chr number, the order of discovery of the locus on that chr (i.e., A, B, etc.) and a sequential number for each modifier at that locus (i.e., M1, M2, M3, etc.).

b Locus supported by a single rare SNP allele in continuous analysis.

Although there was no significant difference in age at onset between the sexes (Figure S3A), male- and female-specific association analyses revealed differences in relative effect size and significance for some modifiers (Figures S3B–S3E; Table 1). This was most evident for the *MSH3/DHFR* locus, where the common modifiers had a far greater effect in females. The sex-specific analysis also revealed 3 new loci (chr 1, 12, and 18) that may contain male-specific modifiers or, since they are tagged by very rare alleles, may be due to statistical outlier effects.

We also performed a transcriptome-wide association study (TWAS) to broadly test association of gene expression and residual age at onset (Gusev et al., 2016). Four genes at 3 loci were significant after Bonferroni correction: *FAN1* (p = 1.9E-22), *MSH3* (1.9E-8), *PMS1* (6.1E-7), and *ASNSD1* (5.3E-6) with later onset associated with increased *FAN1*, *PMS1*, and *ASNSD1* expression and decreased *MSH3* expression. Comparison of the heritability estimation from summary statistics (HESS) regional heritability estimates (Shi et al., 2016) before and after conditioning on expression predicted that 40%, 57%, and 87% of the contribution of *FAN1*, *PMS1*, and *MSH3*, respectively, to the genetic age at onset liability could be explained by *cis* expression effects.

Finally, we performed gene-wide association analysis (Table S2), finding that all significant genes came from regions in Table 1. Pathway enrichment compared to GWA123 again pointed in GWA12345 (and GWA45) to mismatch repair but did not support mitochondrial regulation (Table S3). In 77 DNA repair gene sets (Pearl et al., 2015), the strongest enrichments were related to mismatch repair (Tables S4 and S5). In a broader test of 14,210 pathways with 10 or more gene members, 77 pathways were significant after Bonferroni correction in GWA12345 (Table S6). The top 13 pathways were related to DNA maintenance processes, but some top GWAS genes, such as *FAN1*, *RRM2B*, and *UBR5*, appeared only in the largest, most general pathway (GO 6281: DNA repair). These analyses provide robust evidence for genes involved in DNA maintenance processes in influencing the timing of HD onset. Taken together with the rate-determining property of the uninterrupted CAG repeat, the delineation of multiple DNA maintenance genes as modifiers suggests that these modifiers influence HD age at onset through a DNA-level effect on somatic expansion of the *HTT* CAG repeat.

### Multiple Mismatch Repair Genes Modify HD

**Figure 3:**
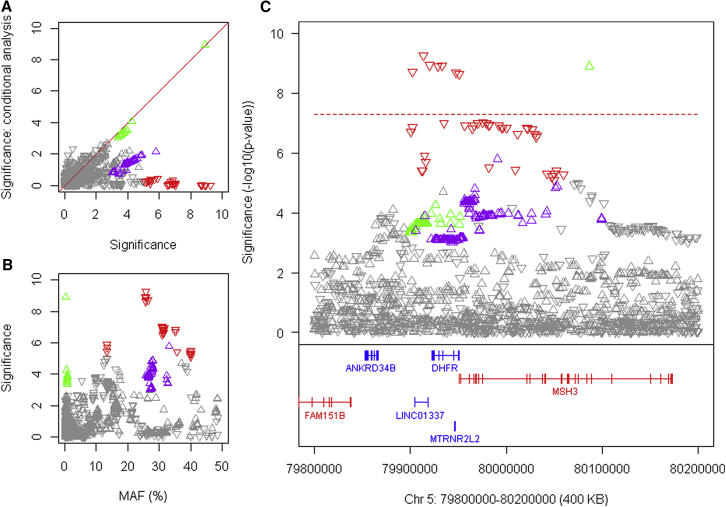
Three HD Onset Modification Signals at *DHFR/MSH3* (A) SNP significance (–log10(p value) in continuous analysis conditioned on the top 5AM1 (red) SNP compared to GWA12345 significance reveals independent modifier haplotypes with p < 1E-5: 5AM2 (green) and 5AM3 (purple). (B) SNP significance compared to MAF. (C) SNP significance relative to genes in the region. See also Figures S4 and S5.

A chr 5 modifier locus centers on *MSH3*, which encodes a DNA mismatch repair protein, and *DHFR*, which produces dihydrofolate reductase, a critical enzyme in determining nucleotide pools. The top SNP tags a frequent onset-hastening modifier effect (haplotype 5AM1) and conditional analyses has revealed 2 additional onset-delaying haplotypes (5AM2 and 5AM3; Figure 3; Table 1). An *MSH3* coding variant has been reported to tag a modifier of a multi-factor progression measure of HD deterioration in the TRACK-HD study (Moss et al., 2017). That variant maps within tandem repeats and is not imputed using HRC data, but a re-examination of our data using the 1000 Genomes as a reference set (Auton et al., 2015) revealed that our 5AM3 haplotype corresponds to the progression modifier in TRACK-HD.

**Figure S4:**
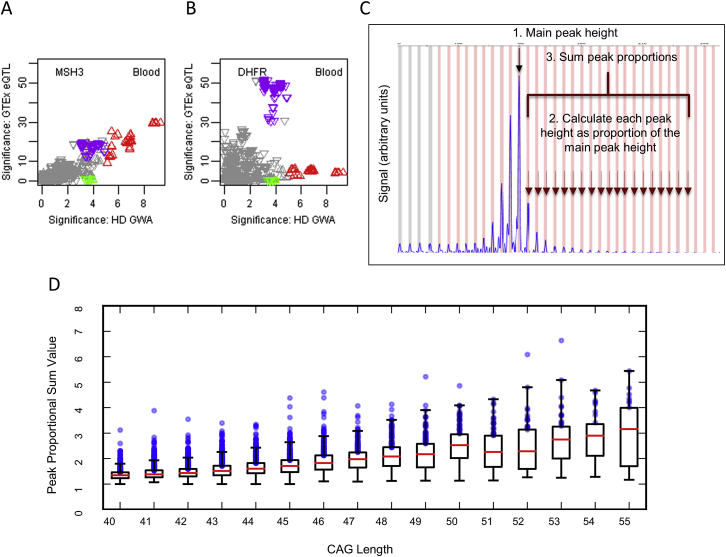
Onset-Hastening Modifier 5AM1 Is Associated with Somatic CAG Repeat Expansion, Related to Figure 3 (A and B): HD modifier GWAS signals in the *MSH3*/*DHFR* region of chr 5 region (x axis, -log10(p value)) were compared to GTEx eQTL signals for *MSH3* and *DHFR* in whole blood (**A**) and (**B**), respectively). Upward and downward triangles represent SNPs whose minor alleles were associated with increased and decreased expression levels of the test gene, respectively. Red, green, and purple triangles represent SNPs tagging modifier haplotypes 5AM1, 5AM2, and 5AM3, respectively, as in Figure 3. (C) Method for calculating the peak proportional sum of *HTT* CAG expansion values from ABI GeneMapper fragment sizing profiles of expanded alleles from our CAG genotyping assay. The bulk of the PCR product for any individual corresponds to the presumptive inherited expanded CAG size and constitutes a floor with respect to which expansion can be examined. PCR products to the left of the main peak are believed to be due largely to PCR artifacts. The peaks to the right of the main peak result from somatic CAG expansions. These are summed and then divided by the size of the main peak to calculate the peak proportional sum of expanded alleles in that individual. The method detects those mosaic individuals with the highest proportion of somatically expanded alleles but is not effective for resolving individuals with reduced somatic expansion. Consequently, it is only applicable to modifier SNPs minor alleles that 1) correspond to blood eQTLs or structural changes, 2) are sufficiently frequent to generate an adequate sample size and 3) are associated with increased somatic expansion (expected to correspond to hastened onset). Of the modifiers in Table 1, only 5AM1 met these criteria. (D) The boxplot shows the peak proportional sum *HTT* CAG repeat expansion values determined for 7,013 GWA12345 subjects, plotted as quartiles (whiskers1.5^∗^IQR) for each CAG repeat size. The proportion of expanded alleles per individual increases with inherited CAG repeat length. The top quartile at each repeat length, representing individuals with the highest proportion of somatic expansions, is distinguished by a wider range of values than the other three quartiles across the CAG repeat lengths. The values for the 1,753 individuals in the highest expansion quartile are plotted as blue circles. Among these individuals, 5AM1-tag SNP rs701383 deviates from Hardy-Weinberg expectation (chi-square 15.80, 2 d.f., p < 0.0004) due to an excess of the A minor allele, associated with hastened onset and increased *MSH3* expression.

**Figure S5:**
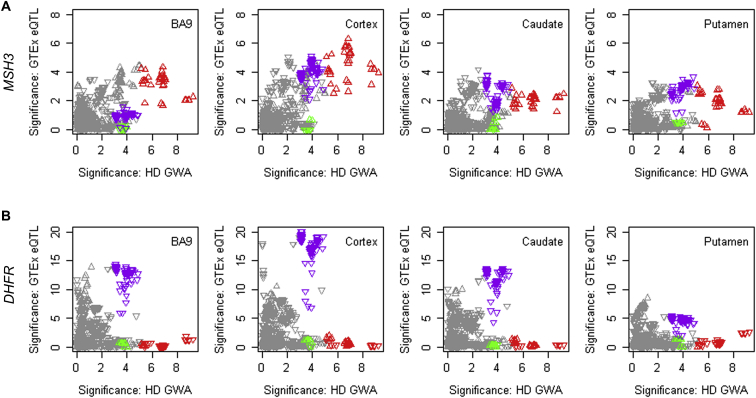
Correspondence between Onset Modifier Signals and GTEx eQTL Signals for *MSH3* and *DHFR*, Related to Figure 3 (A B) HD modifier GWAS signals in the *MSH3*/*DHFR* region of chr 5 region (x axis, -log10(p value)) were compared to GTEx eQTL signals for *MSH3* and *DHFR* (A) and (B), respectively) in BA9, cerebral cortex, caudate and putamen whole blood. Upward and downward triangles represent SNPs whose minor alleles were associated with increased and decreased expression levels of the test gene, respectively. Red, green, and purple triangles represent SNPs tagging modifier haplotypes 5AM1, 5AM2, and 5AM3, respectively, as in Figure 3. There was no sex difference in the expression of *MSH3* in any of the tissues (blood p = 0.555; BA9 p = 0.586; cortex p = 0.993; putamen p = 0.542; or caudate p = 0.054). Interaction between sex and the top 5AM1 SNP in influencing expression was not nominally significant in any tissue (blood p = 0.799; BA9 p = 0.867; cortex p = 0.498; putamen p = 0.985) except caudate (p = 0.025) but the latter significance was eliminated by multiple testing correction. Larger samples sizes will be required for a definitive conclusion.

In GTEx Consortium data, the top 5AM1 SNP alleles correspond strongly to *cis-*eQTLs for increased *MSH3* (but not *DHFR*) expression in blood cells (Figures S3A and S3B). In mice, several mismatch repair genes, including *Msh3*, influence the somatic instability of CAG repeats (Dragileva et al., 2009, Schmidt and Pearson, 2016, Tomé et al., 2013) and in humans, naturally occurring *MSH3* polymorphisms are associated with instability of the non-coding myotonic dystrophy type 1 CTG repeat in blood DNA (Morales et al., 2016). The increased *MSH3* expression from onset-hastening 5AM1 would be predicted to be associated with higher somatic CAG expansion levels based on its suppression in *Msh3* knockout mouse models. We tested this hypothesis in blood using ABI GeneMapper fragment sizing profiles of expanded alleles from our CAG genotyping assay (Figure S4C). For any individual, the bulk of the PCR product corresponds to the presumptive inherited expanded CAG size and constitutes a floor with respect to which expansion can be examined. Those individuals with mosaicism for the highest somatic CAG expansions compared with this inherited size are evident from the increased fraction of larger PCR products detected. Among the 7,013 individuals with suitable traces, there was an inherent increase in expansion with CAG repeat size (Figure S4D) so we identified the 25% (N = 1,753) with the highest proportion of somatic expansions at each repeat length from 40–55 CAGs and examined their genotype at rs701383. This 5AM1 tag SNP deviated significantly from Hardy-Weinberg expectation (chi-square 15.80, 2 d.f., p < 0.0004) due to an excess of the minor A allele (observed 915 GG, 684 AG, 154 AA versus expected 968 GG, 669 AG, 116 AA) indicating that increased *MSH3* expression can be associated with increased somatic expansion. In the brain, the top 5AM1 SNPs correspond less well with *MSH3 cis*-eQTL signals, suggesting that additional factors regulating the locus beyond the steady state may be important for determining CAG expansion (Figure S5). These additional influences may explain the sex difference in the effect of this modifier, since the *cis*-eQTLs do not appear to be sex specific (Figure S5, legend).

In contrast, the rare delayed-onset 5AM2 SNP alleles do not overlap with *cis*-eQTLs for either *MSH3* or *DHFR* (Figure S5) and do not differ in effect size between the sexes, suggesting that 5AM2 may harbor a variant that alters mRNA and/or protein. 5AM3-tagging alleles correspond more robustly with *cis*-eQTLs for decreased *DHFR* expression in cortex, caudate, putamen, and blood than with *MSH3 cis*-eQTLs (Figures S5D and S5E), suggesting potential roles for both *MSH3* and *DHFR* in HD modification.

Three other mismatch repair genes with single modifier effects were also implicated. At *MLH1*, the peak SNP specifies a missense change, I219V, which is considered benign (SIFT: tolerated), but this does not exclude a subtle effect on the activity or interactions of MLH1 in the context of CAG repeat expansion. There was no strong evidence in the TWAS or GTEx Consortium data for an association with altered *MLH1* expression. *PMS1* and *PMS2* (postmeiotic segregation increased 1 and 2, respectively) both encode proteins that form heterodimers with MLH1 in mismatch repair (Pearl et al., 2015). The TWAS suggested a significant effect of *PMS1* expression but no comparably significant expression evidence for *PMS2*. While we have not identified either functional variant, the most parsimonious explanation is that modification acts through these DNA repair genes rather than other genes in these regions.

### *FAN1* Displays Onset-Hastening and -Delaying Haplotypes

**Figure 4:**
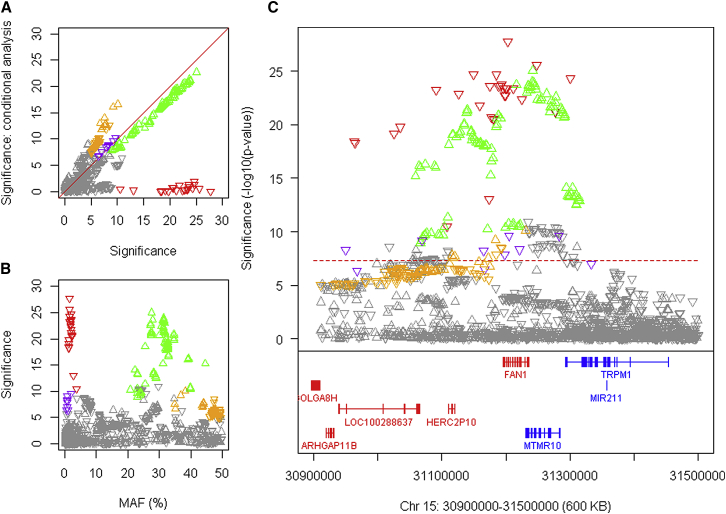
Four HD Onset Modification Signals at *FAN1* (A) SNP significance (–log10(p value) in continuous analysis conditioned on the top 15AM1 (red) SNP compared to GWA12345 significance reveals independent modifier haplotypes: 15AM2 (green), 15AM3 (purple), and 15AM4 (gold). Because SNPs tagging 15AM4 have alleles of close to equal frequency, the direction of the arrow varies depending on whether the minor or major allele is on the 15AM4 haplotype. (B) SNP significance compared to MAF. (C) SNP significance relative to genes in the region. See also Figure S6.

**Figure S6:**
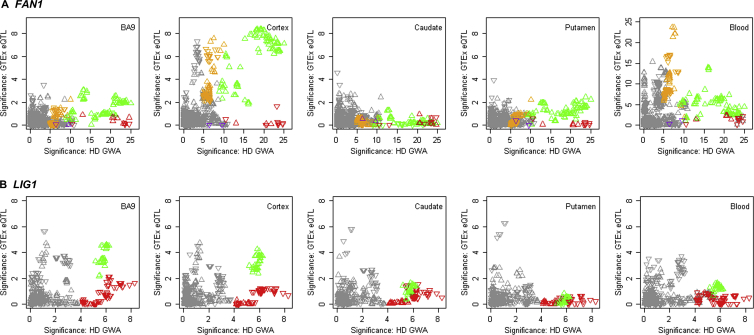
Correspondence between Onset Modifier Signals and GTEx eQTL Signals for *FAN1 and LIG1*, Related to Figures 4 and 5 (A) Chromosome 15 onset modifier signals depicted (as in Figure 4) in red (15AM1), green (15AM2), purple (15AM3), and gold (15AM4) (x axis) were compared to GTEx eQTL signals (y axis) for brain regions and whole blood. Upward and downward triangles represent SNPs whose minor alleles were associated with increased and decreased expression levels of *FAN1,* respectively. Because SNPs tagging 15AM4 have alleles of close to equal frequency, the direction of the arrow varies depending on whether the minor or major allele is on the 15AM4 haplotype. (B) Chromosome 19 onset modifier signals depicted in red (19AM1) and green (19AM2) (x axis) were compared to GTEx eQTL signals (y axis) for *LIG1* expression in brain regions and whole blood. The tag SNP for 19AM3 is too infrequent to appear in GTEx eQTL results. Upward and downward triangles represent SNPs whose minor alleles were associated with increased and decreased expression levels of *LIG1,* respectively.

GWA12345 replicated the opposing effects of an infrequent onset-hastening modifier (15AM1) and a frequent onset-delaying modifier (15AM2) from GWA123 revealed two novel modifier haplotypes, 15AM3 and 15AM4 (Figure 4; Table 1), and pointed directly to *FAN1* as the source of HD modification. *FAN1* encodes a nuclease with involvement in interstrand DNA crosslink (ICL) repair (Smogorzewska et al., 2010) that is also recruited to stalled replication forks, physically interacts with MLH1, and is needed for homologous recombination but not double-strand break resection (Cannavo et al., 2007, Lachaud et al., 2016, MacKay et al., 2010). 15AM1 and 15AM3 are both tagged by SNP alleles that specify missense variants, R507H and R377W, respectively, both predicted to be deleterious by SIFT. The former has been associated with karyomegalic interstitial nephritis, a recessively inherited disease caused by loss of FAN1 function (Bastarache et al., 2018). Tag SNPs for 15AM2 and 15AM4 correspond with *cis*-eQTLs for increased expression in cortex (Figure S6A). No other gene in the region shows a missense variant or correspondence between any cortex, caudate, or putamen *cis*-eQTLs and modifier association signals. Thus, our data point to *FAN1* as the source of the modifier effects, with reduced function hastening onset and increased expression delaying onset. Notably, lowering *FAN1* expression in mammalian cells and patient-derived induced pluripotent stem cells (iPSCs) induced *HTT* CAG expansions (Goold et al., 2018), and preliminary findings have indicated a similar effect of FAN1 deficiency in an HD knockin mouse model (J.L. and M.E.M., unpublished results). Inactivation of *Fan1* induced somatic expansion of a CGG repeat in a mouse Fragile X syndrome model (Zhao and Usdin, 2018), indicating that the impact of *FAN1* variation can extend to other non-CAG, non-coding repeat diseases.

### *LIG1* Displays 3 Modifier Haplotypes

**Figure 5:**
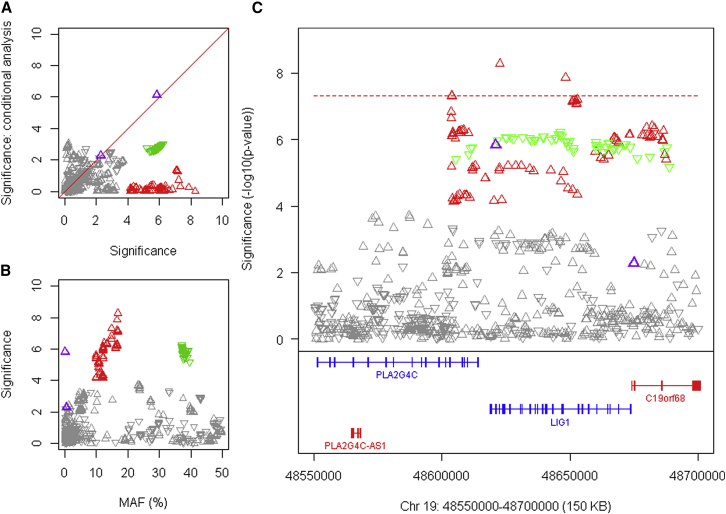
Three Onset Modification Signals at *LIG1* (A) SNP significance (–log10(p value) in continuous analysis conditioned on the top 19AM1 (red) SNP compared to GWA12345 significance reveals independent modifier haplotypes: 19AM2 (green) and 19AM3 (purple). (B) SNP significance compared to MAF. (C) SNP significance relative to genes in the region. See also Figure S6.

*LIG1*, on chr 19, encodes an ATP-dependent DNA ligase that seals DNA nicks during replication, recombination, and a variety of DNA damage responses (Howes and Tomkinson, 2012). This locus revealed 2 common modifier haplotypes with opposing effects on HD onset (Figure 5; Table 1) and a third rare modifier haplotype (19AM3) that is strongly onset delaying. 19AM3 is tagged by a LIG1 missense change, K845N, predicted to be deleterious by SIFT, suggesting that reduced activity and/or altered interactions of LIG1 protein may suppress CAG repeat expansion. Conversely, the onset-hastening 19AM2 is associated with increased expression of LIG1 in cortex and BA9 (Figure S6B), consistent with increased CAG repeat instability due to exogenous overexpression of LIG1 in human cells (López Castel et al., 2009). The mechanism by which 19AM1 acts is not clear.

### Other Modifiers Are Not Directly Involved in DNA Maintenance

**Figure 6:**
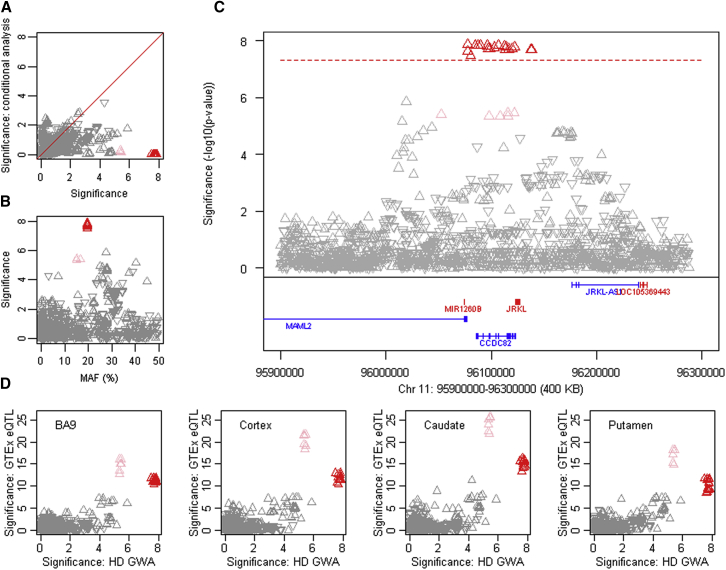
An Onset Modification Signal at *CCDC82* (A) SNP significance in dichotomous analysis conditioned on the top 11AM1 (red) SNP compared to GWA12345 significance reveals the presence (with subsequent analyses) of a related haplotype (pink triangles). (B) SNP significance compared to MAF, revealing a slight difference for tag SNPs of the closely related haplotypes. (C) SNP significance relative to genes in the region. (D) SNP significance in dichotomous analysis is compared to *cis*-eQTL signals for *CCDC82* in GTEx consortium data for prefrontal cortex BA9, cortex, caudate, and putamen. In this case, the direction of the triangle indicates association of the minor allele with increased (upward) or decreased (downward) expression. Both related haplotypes (red and pink) show correlation with *cis*-eQTL SNPs associated with increased expression of *CCDC82* in all 4 brain regions. See also Figure S7.

**Figure S7:**
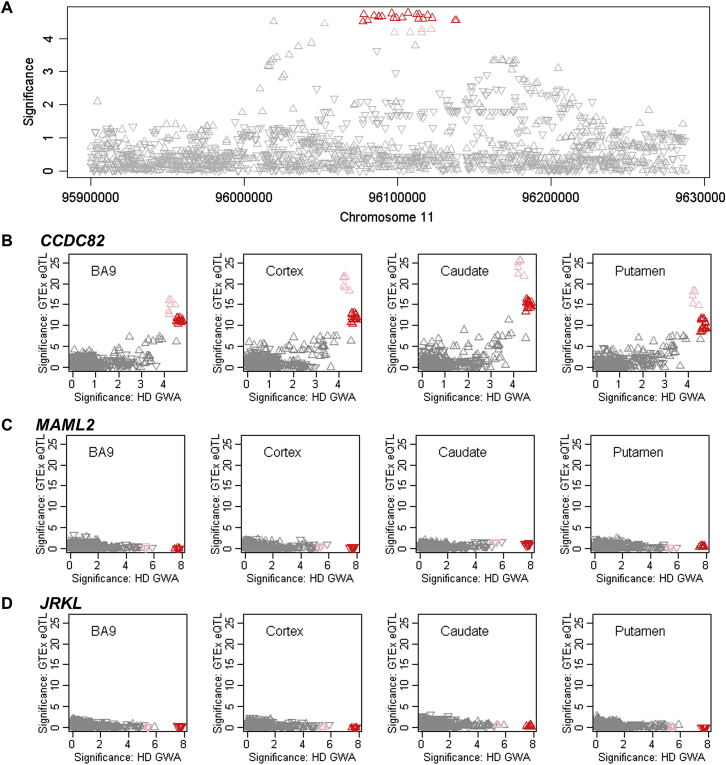
Association Signals in Continuous Phenotype Analysis for Chromosome 11 Region and Correspondence to GTEx eQTL Signals, Related to Figure 6 (A) Association analysis for SNPs in the chromosome 11 region using the continuous phenotype. (B). These continuous phenotype analysis results were compared to GTEx eQTL signals for *CCDC82* in brain regions. (C and D) GTEx eQTL signals for *MAML2* (C) and *JRKL* (D) in selected brain regions (y axis) were compared to chromosome 11 onset modifier signals based on the dichotomous phenotype (from Figure 6). Red and pink triangles represent SNPs marking the 11AM1 and a closely related haplotype.

The other modifier loci could theoretically act indirectly on DNA maintenance processes or represent independent modification mechanisms. *RRM2B*, encoding the small subunit of a p53-inducible ribonucleotide reductase, and *UBR5*, specifying an E3 ubiquitin-protein ligase, are candidates on chr 8. On chr 11 (Figure 6), there is correspondence between SNP alleles associated with delayed onset and *cis*-eQTL alleles for increased expression of *CCDC82* in cortex, caudate, and putamen but no such relationship for other genes in the region (Figure S7), suggesting that increased CCDC82 acts to delay onset. Relatively little is known concerning this coiled-coil domain protein, beyond it being reported as a substrate for ATM-dependent phosphorylation in response to H_2_O_2_ treatment (Kozlov et al., 2016). Perhaps most intriguing is a second chr 5 locus, where a single SNP, rs79727797, supported by both continuous and dichotomous analysis, is in an intron of *TCERG1*, which encodes a nuclear regulator of transcriptional elongation and pre-mRNA splicing. *TCERG1* was proposed as a potential HD modifier based on studies of its polymorphic (Gln-Ala)_n_-encoding repeat, prompted by the protein’s interaction with huntingtin (Holbert et al., 2001). The genome-wide significant GWA signal suggests that more detailed study of this locus is warranted.

## Discussion

Our observations support a model in which the rate at which HD manifestations emerge, leading to clinical diagnosis, is determined not by length-dependent polyglutamine toxicity, but by length-dependent somatic expansion of the CAG repeat in critical target cells. Mouse HD knockin models display length-dependent somatic expansion of the CAG repeat throughout the brain, but most prominently in the striatum (Lee et al., 2010, Wheeler et al., 1999), and this process is modified by DNA maintenance genes (Dragileva et al., 2009, Pinto et al., 2013, Wheeler et al., 2003). In humans, somatic CAG repeat expansion has been observed in HD and more broadly in other polyglutamine disorders (López Castel et al., 2010), arguing that somatic expansion may be central to all polyglutamine diseases, and potentially to other diseases with repeat expansion outside coding sequence (Nelson et al., 2013). Somatic CAG expansion has been demonstrated in HD post-mortem brains (Kennedy et al., 2003, Swami et al., 2009) with the earliest onset individuals at any inherited CAG repeat length showing the largest somatic expansions, consistent with length-dependent CAG expansion being the rate driver for onset. Like CAA interruptions in *HTT*, CAT interruptions of the *ATXN1* CAG repeat in four spinocerebellar ataxia 1 subjects supported better tracking of age at onset with the uninterrupted CAG repeat size, but, because CAT specifies histidine, this was interpreted as an effect of polyglutamine length (Menon et al., 2013). Analysis in follow-up to GWA123 provided evidence of DNA maintenance processes also modifying timing of onset in a collection of spinocerebellar ataxias, supporting a shared property of expanded CAG repeats as the critical driver of age at onset in multiple polyglutamine diseases (Bettencourt et al., 2016).

A theoretical mathematical framework has been proposed previously to explain the emergence of disease symptoms broadly in trinucleotide repeat diseases based upon somatic expansion of the underlying repeats (Kaplan et al., 2007). This computational approach argued against continuous toxicity of the mutant allele and relied instead on somatic expansion to a critical threshold repeat length as causing the dysfunction of vulnerable cells. In addition to being consistent with our GWAS findings, this mathematical model also explains the lack of earlier onset in individuals with two mutant and no normal *HTT* alleles (Figure S2F) and of similar cases in other polyglutamine diseases (Gusella and MacDonald, 2000, Lee et al., 2012b). The model postulates a rapidly increasing probability of further somatic expansion as the CAG repeat length increases. The larger of the two expanded CAG alleles (or the first to expand if equal) would predominate as its more rapid further expansion would out-pace the remaining allele in reaching the critical threshold length to trigger toxicity. However, this model does not speak to the nature of the toxicity driver, i.e., to what causes the actual damage to vulnerable neurons once the size threshold is reached. An above-threshold expanded CAG repeat might trigger polyglutamine toxicity, but there is no definitive demonstration that this is the primary cause of cellular damage in human HD, so one must also consider other mechanisms that have been proposed at the level of the *HTT* mRNA (Martí, 2016), splicing (Sathasivam et al., 2013), translation (Gao et al., 2017), or even via an effect at the DNA level on chromatin domains (Bruneau and Nora, 2018). Indeed, the discovery that spinocerebellar ataxia 12 is caused by a similar transcribed CAG repeat expansion at *PPP2R2B* that does not produce polyglutamine (Cohen and Margolis, 2016), argues that other mechanisms must also be considered as potential toxicity drivers in the various CAG repeat diseases. Each disease may also involve different threshold somatic CAG expansion lengths in different target cell types to produce disease onset. Ultimately, the disease presentation in any of the trinucleotide repeat disorders may depend on a combination of (1) the rate at which CAG repeats expand in the critical target cell type(s), (2) the degree to which modifiers act to influence expansion rate, (3) the threshold somatic expansion size for cellular damage to occur, (4) the mechanism, polyglutamine toxicity or other, by which that somatically expanded repeat causes the cellular damage, and (5) the degree to which the damage mechanism is influenced by modifiers. Indeed, these factors may vary even within a single disease, since the 15AM1 and 15AM2 modifiers showed differential influences on phenotypes in premanifest HD individuals, suggesting differential modification in target cell types critical to each phenotype (Long et al., 2018). In addition, duration of manifest HD from onset to death is independent of CAG repeat length, suggesting either that, once cellular damage has reached a critical level, HD progression is more dependent on other physiological and/or clinical factors or that different cell types are involved in the progression of HD after onset (Keum et al., 2016). These considerations emphasize the complexity of defining pathogenic mechanisms and their phenotypic correlates in this late-onset disorder, since different cell types display different rates of CAG expansion (Lee et al., 2010) and, conceivably, differences in threshold lengths for initiation of cellular damage, susceptibility to modifiers, and/or toxicity drivers.

The *HTT* CAA-loss and CAACAG-duplication alleles mark a very small proportion of HD chrs, but implementation of a DNA sequence-based CAG repeat assay will be important for avoiding molecular diagnostic misinterpretations at the borders of high normal (27–35 CAGs), reduced penetrance (36–39 CAGs), and full penetrance (>39 CAGs) size ranges. As additional variations of the CAG emerge, testing should proceed cautiously since larger sample sizes and phenotypic detail will be needed to predict outcomes for the <5% of individuals with non-canonical sequences. From the research perspective, rare *HTT* alleles may be particularly informative for guiding therapeutic strategies. For example, discovery of an allele with a CAA (or other codon) interruption centrally located within a long *HTT* CAG repeat that fails to cause HD because neither flanking CAG repeat exceeds 35 CAGs would support a therapeutic strategy aimed at introducing such interruptions into *HTT*.

The notion that the rate driver for disease onset in HD and other repeat disorders can be modified by DNA maintenance processes also provides a prime target for developing broadly applicable therapies to prevent or delay disease onset by intervening prior to action of the toxicity driver(s) in each disorder. DNA maintenance genes have been investigated mainly in cancer cells, where inactivating mutations may cause cancer initiation or progression. Interestingly, inactivation of mismatch repair genes, which causes dinucleotide repeat instability in colon cancer, suppresses trinucleotide repeat instability in mouse models, suggesting distinct mechanisms (Boland and Goel, 2010, Usdin et al., 2015). While the association with cancer dictates a need for caution in manipulating these pathways to prevent neurodegenerative diseases, human genetic evidence demonstrates that DNA maintenance gene activities can vary over a relatively wide range without major deleterious effects. Each modifier gene must influence HD by virtue of naturally occurring variation in either activity, expression level, or regulation, yet none has emerged as a risk factor in a cancer predisposition GWAS (GWAS Catalog: https://www.ebi.ac.uk/gwas/). For modifiers whose reduced activity is associated with delayed onset or increased expression with hastened onset, a goal of treatment would be to reduce the activity or level of the target protein sufficiently to achieve an even greater effect than occurs due to naturally occurring modifiers. For example, our findings with modifier 5AM1 suggest that reducing *MSH3* expression levels or activity would inhibit somatic expansion of the *HTT* CAG repeat. Population data show that *FAN1*, *MSH3*, *PMS1*, *PMS2*, and *LIG1* (pLI < 0.02) are all tolerant to loss-of-function variants (Karczewski et al., 2017), which are not rapidly removed by selective pressure (Kosmicki et al., 2017). Thus, the activities of each of these proteins can vary over at least a 2-fold range (wild type versus loss-of-function heterozygote) in normal individuals without producing strong negative selection. By contrast, *HTT* (pLI = 1.00) is among the most loss-of-function intolerant genes, showing far fewer naturally occurring inactivating mutations than would be expected, presumably due to their elimination by an as yet undetermined selective pressure.

Our results also point to other potential modifier genes (e.g., *RRM2B/UBR5*, *CCDC82*) that could conceivably influence the toxicity mechanism by which a somatically expanded repeat that exceeds its critical threshold precipitates cellular damage. Identification of the mode of action of such genes could provide an entrée into aspects of disease progression whose rate is not driven by the length of the CAG repeat. After HD clinical onset, the disease progresses with deterioration in motor, cognitive, and, in many cases, psychiatric domains, in parallel with neurodegeneration and loss of body mass. That duration of the manifest disease phase is independent of the inherited *HTT* CAG length (Keum et al., 2016) suggests that some disease changes after onset involve processes or cell types different from those that drive the rate of onset. The power of the GWAS approach to detect genetic modifiers that act before the onset of HD has been amply demonstrated. While the sample size can be further increased to dig deeper into this onset modifier pool and to support the inclusion of modifier SNPs in optimizing clinical trial power and/or design, the strategy can now also be applied broadly to define modifiers that influence disease progression. To this end, we are pursuing a variety of approaches to define disease stages and corresponding phenotypes for GWA analysis, particularly time-interval phenotypes, like disease duration, that are less dependent on or independent of CAG repeat length. We expect that this human genetics strategy will identify modifiers that help to delineate the toxicity driver(s) and ultimately will successfully illuminate each phase of HD, from premanifest to eventual death. This approach has the potential to highlight specific targets for therapeutic intervention at different disease stages, allowing a stratified approach to treatment over the protracted, complex disease course.

## STAR★Methods

### Key Resources Table

**Table:** 

REAGENT or RESOURCE	SOURCE	IDENTIFIER
**Critical Commercial Assays**		

InfiniumOmniExpressExome-8v1-3_A.	Illumina (https://www.illumina.com/products/by-type/microarray-kits/infinium-omni-express-exome.html)	
Multi-EthnicGlobal-8_A1 arrays	Illumina (https://www.illumina.com/products/by-type/microarray-kits/infinium-multi-ethnic-global.html)	
Nextera XT Index Kit v2 Set A (96 indexes, 384 samples)	Illumina	Cat# FC-131-2001
Nextera XT Index Kit v2 Set B (96 indexes, 384 samples)	Illumina	Cat# FC-131-2002
Nextera® XT Index Kit (24 indexes, 96 samples)	Illumina	Cat# FC-131-1001
MiSeq® Reagent Kit v3 (600 cycle)	Illumina	Cat# MS-102-3003
QIAGEN LongRange PCR Kit (250)	Qiagen	Cat# 206403
AmpliTaq Gold DNA polymerase with BufferII, MgCl2 12x 250units	Life Technologies	Cat# N8080245

**Deposited Data**		

Common Mind Consortium gene expression data	https://www.synapse.org/#!Synapse:syn2759792/wiki/69613	
Gene Ontology (GO)	http://geneontology.org/ (Gene Ontology Consortium, 2015)	
Kyoto Encyclopedia of Genes and Genomes (KEGG)	https://www.genome.jp/kegg/ (Kanehisa et al., 2016)	
Mouse Genome Informatics (MGI),	http://www.informatics.jax.org/ (Eppig et al., 2015)	
National Cancer Institute (NCI) Pathway Interaction Database	http://www.ndexbio.org/#/network/47eb8891-86da-11e7-a10d-0ac135e8bacf (Schaefer et al., 2009)	
Protein ANalysis THrough Evolutionary Relationships (PANTHER),	http://www.pantherdb.org/ (Mi et al., 2013).	
BioCarta	(Nishimura, 2001)	
Reactome	https://reactome.org/ (Fabregat et al., 2016)	
The Haplotype Reference Consortium	http://www.haplotype-reference-consortium.org/ (McCarthy et al., 2016)	
1,000 Genomes Project Phase 3 data	http://www.internationalgenome.org/ (Auton et al., 2015)	
Genotype-Tissue Expression (GTEx) project	https://gtexportal.org/home/ (dbGaP: phs000424.v7.p1)	
Simons Simplex Collection Whole Genome Sequence Data	https://www.sfari.org/resource/sfari-base/	
Summary statistics from this study	https://datadryad.org/resource/doi:10.5061/dryad.5d4s2r8	

**Oligonucleotides**		

HD-1 5’ATGAAGGCCTTCGAGTCCCTCAAGTCCTTC3’	(Warner et al., 1993)	
HD-3 5’GGCGGTGGCGGCTGTTGCTGCTGCTGCTGC3’	(Warner et al., 1993)	
HTT ms_hd_f *TCGTCGGCAGCGTCAGATGTGTATAAGAGACAG*ATG AAGGCCTTCGAGTCCC	This paper	N/A
HTT ms_hd_r *GTCTCGTGGGCTCGGAGATGTGTATAAGAGACA*GGG CTGAGGAAGCTGAGGA	This paper	N/A

**Software and Algorithms**		

Birdsuite	https://www.broadinstitute.org/birdsuite/birdsuite	
Michigan Imputation Server	https://imputationserver.sph.umich.edu/index.html#! (Das et al., 2016)	
PLINK	http://zzz.bwh.harvard.edu/plink/ (Purcell et al., 2007)	
Genome-wide Efficient Mixed Model Association (GEMMA)	http://www.xzlab.org/software.html (Zhou and Stephens, 2012)	
METAL	https://genome.sph.umich.edu/wiki/METAL_Documentation (Willer et al., 2010)	
GenABEL R package (version 1.8-0)	https://cran.r-project.org/src/contrib/Archive/GenABEL/GenABEL_1.8-0.tar.gz (Aulchenko et al., 2007)	
FUSION	(Gusev et al., 2016)	
MAGMA	(de Leeuw et al., 2015)	
ALIGATOR	(Holmans et al., 2009)	
GeneMapper v3.7 and v5.0	Applied Biosystems	
TREDPARSEv0.7.8: HLI Short Tandem Repeat (STR) caller	https://github.com/humanlongevity/tredparse (Tang et al., 2017)	
SciPy: Open Source Scientific Tools for Python	http://www.scipy.org	
Samtools(v1.7)	http://www.htslib.org/ (Li et al., 2009)	
Knime Analytics Platform (v3.6.2)	https://www.knime.com/knime-software/knime-analytics-platform	
R (v3.3.1, v 3.3.3 and v3.5.0)	https://www.r-project.org/	
SIFT (Sorting Intolerant from Tolerant)	Implemented through the Variant Effect Predictor, https://useast.ensembl.org/Tools/VEP (Vaser et al., 2016)	

**Other**		

ABI 3730XL DNA Analyzer	Applied Biosystems	N/A
MiSeq	Illumina	N/A
Tape Station 2200	Agilent	N/A
LightCycler480	Roche	N/A

### Lead Contact and Materials Availability

Further information and requests for resources and reagents should be directed to and will be fulfilled by the Lead Contact, James F. Gusella, Ph.D. (gusella@helix.mgh.harvard.edu). Data involving human subjects will be shared with qualified investigators given their institutional assurance that subject confidentiality will be ensured and that there will be no attempt to discover the identity of any human subject.

### Experimental Model and Subject Details

Patient consents and the overall study were reviewed and approved by the Partners HealthCare Institutional Review Board. We analyzed genetic data from 9,064 HD subjects including 4,271 available from a previous GWA study (Genetic Modifiers of Huntington’s Disease (GeM-HD) Consortium, 2015) and 4,793 genotyped and QC-passed in the current study. Phenotypic data for the newly analyzed subjects were made available by the ENROLL-HD platform (https://www.enroll-hd.org/) and the EHDN Registry study (http://www.ehdn.org/), who both approved our procurement of the DNA of these subjects from their repositories at BioRep Inc. (Milan, Italy). Enroll-HD is a global clinical research platform designed to facilitate clinical research in Huntington’s disease. Core datasets are collected annually from all research participants as part of this multi-center longitudinal observational study. Data are monitored for quality and accuracy using a risk-based monitoring approach. Registry is multi-center, multi-national observational study that has been described (Orth et al., 2010). All sites are required to obtain and maintain local ethical approval. For this study, HD age at onset was defined as the age at which significant motor signs are noted by a rater expert in the Unified Huntington’s Disease Rating Scale (UHDRS). In the small minority of cases where no expert rater estimate was available, we used the estimate provided by family members of the HD subject.

### Method Details

#### Genome-wide SNP genotyping

DNA samples for GWA4 (Registry subjects) and GWA5 (Enroll_HD subjects) were genotyped by InfiniumOmniExpressExome-8v1-3_A (https://www.illumina.com/products/by-type/microarray-kits/infinium-omni-express-exome.html) and Multi-EthnicGlobal-8_A1 arrays (https://www.illumina.com/products/by-type/microarray-kits/infinium-multi-ethnic-global.html), respectively. Genotyping and genotype calling based on the Birdsuite algorithm (https://www.broadinstitute.org/birdsuite/birdsuite) were performed at the Broad Institute. We performed quality control (QC) analysis for each GWA independently in order to generate a dataset for genotype imputation. Briefly, we identified HD subjects with European ancestry (based on comparison of study subjects to HapMap samples) and subsequently excluded SNPs that showed genotyping call rate < 95% or minor allele frequency < 1%.

#### Genotype imputation and quality control

Each QC passed dataset, including those obtained for the prior GWA1, GWA2 and GWA3 (Genetic Modifiers of Huntington’s Disease (GeM-HD) Consortium, 2015), was further subjected to additional QC analyses as part of the imputation process by the Michigan Imputation Server (https://imputationserver.sph.umich.edu/index.html#!) (Das et al., 2016). Briefly, SNPs tagged due to strand mismatch were excluded, as were any samples with a low genotyping call rate (< 50% of SNPs) for any 20 Mb chunk of the genome. Subsequently, haplotype phasing was performed by EAGLE (v2.3.2) and genotypes were imputed separately for GWA1-5 using MINIMAC via the Michigan Imputation Server, using the Haplotype Reference Consortium data (Version r1.1 2016) (http://www.haplotype-reference-consortium.org) (McCarthy et al., 2016) as the reference panel. Post-imputation, we further excluded SNPs with 1) imputation R square value < 0.5 in any of the GWA datasets, 2) call rate < 100%, 3) Hardy-Weinberg equilibrium p value < 1E-6 except the chromosome 4:1-5,000,000 region, or 4) minor allele frequency < 0.1%. These quality control filters generated a total of 10,986,607 imputed SNPs for 9,064 HD subjects with age at onset data for genetic association analysis.

#### *HTT* CAG repeat genotyping assay

*HTT* uninterrupted CAG repeat size was estimated using a modified PCR amplification assay (Warner et al., 1993), adapted for the ABI3730XL DNA sequencer in a 96 well plate format. Each plate includes reactions for genomic DNA *HTT* CAG size standards, previously sequenced and known to have different uninterrupted CAG repeat lengths in the normal and expanded CAG repeat ranges. Reactions are in a total volume of 10 ul containing 1.25 mM MgCl_2_ (Applied Biosystems), 1X Buffer II (Applied Biosystems), 0.05 U Amplitaq Gold (Applied Biosystems), 0.25 mM dNTPs (GE Healthcare), 1.2 ul of DMSO (Sigma), 0.125 uM each primer with 80 ng of genomic DNA. The PCR primers (forward primer labeled with 6FAM, reverse primer is tailed) are: Forward primer (HD-1) 5′ ATGAAGGCCTTCGAGTCCCTCAAGTCCTTC 3′ and Reverse primer (HD-3) 5′ GGCGGTGGCGGCTGTTGCTGCTGCTGCTGC 3′. The PCR amplification cycles are: denaturation at 94°C for 4 minutes, thirty-five cycles of denaturation at 94°C for 30 s, annealing at 65°C for 30 s, and extension at 72°C for 45 s, with a final extension at 72°C for 10 minutes and hold at 15°C. The PCR amplification products are loaded onto an ABI 3730XL DNA Analyzer (36 cm array, POP-7 Polymer, standard fragment analysis conditions) along with an internal size standard where 0.8 ul PCR product is loaded in 9.4 ul Hi-Di Formamide (Applied Biosystems), with 0.1 ul GeneScan 500 LIZ (Applied Biosystems). The resulting .fsa files are analyzed with GeneMapper v5.0 (Applied Biosystems) software and the CAG repeat allele sizes are estimated relative to the fragment sizes of the *HTT* CAG repeat genomic DNA standards. By convention, the CAG repeat alleles assigned to each sample are the highest peak-signal (main peak) in the normal and the expanded CAG repeat range.

#### Somatic *HTT* CAG repeat expansion analysis

We classified subject blood cell DNA samples with respect to somatic CAG repeat expansion using the *HTT* CAG genotyping assay output of the GeneMapper v5.0 software (Applied Biosystems). This is not a single molecule assay but rather a bulk measure that has a floor at the inherited CAG repeat size. For a given individual, the majority of PCR products are nested around the peak signal representing the main CAG repeat size, reflecting PCR stutter inherent in the assay that masks small biological variation in CAG repeat size. However, individual samples may display PCR products at lengths detectably greater than nested bulk PCR products. These rarer products represent somatically expanded CAG repeats present in the individual. Consequently, the utility of this assay is to identify those individuals with the highest proportion of such somatic expansions, but it is not a sensitive discriminator for the majority of samples. From the GeneMapper ‘sample plot view’, a peak data table was exported for all peaks (in .txt format) containing the following information: sample name, called CAG allele, peak size in bp, peak height, area under the peak and data point/scan number of the highest point of the peak. Using the assigned main expanded CAG allele and peak size in bp data, a linear regression was performed, on a per plate basis, to assign a CAG length to all expanded peaks. Linear regression was fit using the linregress function from the SciPy Python library Stats module (https://www.scipy.org/) according to the model: *cag*_*i*_ = β_0_ + β_1_ *size*_*i;*_ where cag and size are the assigned main expanded CAG allele and its measured fragment size, respectively. The size data for each sample was then transformed to a CAG repeat length by using the intercept and slope from the linear regression. For peaks whose sizes transform to the same CAG length, the larger peak height was taken. The data were filtered using an upper size threshold of 500 bp and minimum peak height threshold of 50 RFU (relative fluorescent units). To calculate the proportion of expansion products for each sample, the expanded peaks were then transformed to a proportion relative to the height of the main CAG-allele assigned peak for that sample. The proportion of expansion products was then expressed as the sum of the peak-proportions, yielding the “peak proportional sum value” for that sample.

#### *HTT* MiSeq DNA Sequencing

DNA sequencing of *HTT* exon 1 CAG repeat and adjacent region in genomic DNA samples was accomplished using the Illumina MiSeq Adapted Metagenomics 16S Targeted Resequencing Protocol Library Preparation guide (Part # 15044223 Rev. B) using locus specific primers. The Step 1 oligonucleotide primer pair (PCR 1) comprising the forward and reverse *HTT* CAG target sequences and Illumina Adaptor sequence (in italicized text) was:

ms_hd_f TCGTCGGCAGCGTCAGATGTGTATAAGAGACAGATGAAGGCCTTCGAGTCCC

ms_hd_r *GTCTCGTGGGCTCGGAGATGTGTATAAGAGACAG*GGCTGAGGAAGCTGAGGA

The Step 1 PCR reaction conditions in a total reaction volume of 20 ul, comprise 1X Buffer LongRange (QIAGEN), 1X Q-Solution (QIAGEN), 500 uM dNTP (QIAGEN), 0.25 uM each forward and reverse primer, 2 units of LongRange Enzyme (QIAGEN), and 20 ng of genomic DNA. PCR amplification cycles were: initial denaturation at 93°C for 3 minutes, thirty-three cycles of denaturation at 93°C for 30 s, annealing at 60°C for 30 s, and extension at 68°C for 90 s, and hold at 15°C until bead clean up. The Step 2 PCR reaction conditions, in a total reaction volume of 50 ul, comprise 1X Buffer LongRange (QIAGEN), 1X Q-Solution (QIAGEN), 500 uM dNTP (QIAGEN), 0.8 uM each forward and reverse primer, 2 units of LongRange Enzyme (QIAGEN), and 5 ul of Step 1 PCR product, with the following cycle parameters: initial denaturation at 93°C for 3 minutes, eight cycles of denaturation at 93°C for 30 s, annealing at 60°C for 30 s, and extension at 68°C for 90 s, hold at 15°C until bead clean up. The Step 2 PCR amplification products were cleaned up using a 1X bead to PCR product ratio. The libraries were pooled in sets of 24, 96, or 192 and run on the MiSeq at concentrations 8 - 15 pM with 10% PhiX spike-in of the same concentration for a 2x300 bp read. The MiSeq output in FASTQ format was utilized for analysis to determine the size of uninterrupted CAG repeat and adjacent DNA sequence.

#### *HTT* uninterrupted CAG repeat length and adjacent triplet repeat DNA sequence

The length of the uninterrupted CAG repeat and the adjacent sequence were determined from #1) MiSeq, as well as from #2) whole genome sequence data. For #1) the 300 bp paired-end read MiSeq data (FASTQ format) were analyzed as follows. Each sequence read pair was processed using Python (v3.5.1). For the forward strand profiling began at the first instance of a CAGCAGCAG 9-mer (5′end of the uninterrupted CAG repeat tract) and continued across each successive trinucleotide repeat until a CAGCTTCCT 9-mer (3′ end of the polymorphic triplet repeat tract) was encountered or until the end of the read was reached. The read that is antisense to the forward strand was reverse-complemented and was profiled in the same manner. If the uninterrupted CAG repeat tract and adjacent triplet repeat structure matched on both mates of a read pair, then they were aggregated and counted. Unmatched forward- and reverse-strand reads were discarded. The uninterrupted CAG repeat allele was assigned (Python v3.5.1), using the distribution of uninterrupted CAG repeat sizes of the profiled structures, to construct a CAG repeat genotype for each sample by identifying the two most frequent CAG peaks. The highest frequency profiled structures that contained each uninterrupted CAG repeat allele were then used to create a complete genotype (both alleles) for the sample that encompassed both the pure CAG tract and the adjacent triplet repeat sequence. For #2), as part of a separate study of genetic defects in neurodevelopmental disorders, we have been investigating *HTT* as a candidate neurodevelopmental gene (Rodan et al., 2016) in the Simons Simplex Collection whole-genome sequence data (https://www.sfari.org/resource/simons-simplex-collection/). The CRAM files were converted to BAM file format using samtools 1.7. (Li et al., 2009). The TREDPARSEv0.7.8 software package (Tang et al., 2017) was utilized in fullsearch mode in Python v2.7.x, to assign the length of the uninterrupted CAG tract on both alleles. To determine the adjacent sequence, the reads mapping to *HTT* exon 1 were subset from the alignment (samtools 1.7). Each individual read was searched for a 9-mer of CAGCTTCCT, relative to the forward strand. The DNA sequence of the read was then read in reverse (3′ to 5′) until 3 consecutive CAG repeats (CAGCAGCAG) were found (Python 3.5.1). All discovered sequences were then aggregated and counted and only those with 3 consecutive CAG repeats at the 5′ end and the 9-mer at the 3′ end were taken as complete. Complete sequences that represent 14% or less of the aggregated reads were filtered out of further analysis. Samples with a single complete sequence were assigned as homozygotes and samples with two different complete sequences were assigned as heterozygotes. Based upon direct examination of the sequence reads, the length of the uninterrupted CAG repeat was that assigned by TREDPARSE for canonical chromosomes and differed by 2 for the non-canonical chromosomes based upon the use by TREDPARSE of the canonical allele in the human genome reference sequence.

### Quantification and Statistical Analysis

#### Genome-wide association study (GWAS) analysis using the continuous phenotype in meta-analysis and combined analysis

For each study subject, age at onset of diagnostic motor signs and CAG repeat size based on the genotyping assay were used to calculate residual age at onset, representing years of deviation from the expectation. For example, a HD subject with a residual age at onset of +5 indicates an individual who developed motor symptoms 5 years later than expected (compared to the majority of HD subjects) considering their CAG repeat length. We primarily analyzed HD subjects carrying 40-55 CAG repeats to minimize the levels of inaccuracy in calculating the residual age at onset. In addition, dichotomized phenotype analysis was also performed to detect statistical artifacts (see next section). To determine whether our previous association analysis using GWA123 data were replicated, we performed combined analysis of the GWA4 and GWA5 datasets. The residual age at onset of motor symptoms as the continuous dependent variable was modeled as a function of minor allele count of the test SNP, sex, and the first 4 principal component values from the genetic ancestry analysis in a linear mixed effect model with relationship matrix using GEMMA (version, 0.94 beta) (http://www.xzlab.org/software.html) (Zhou and Stephens, 2012). Subsequently, we performed meta-analysis to summarize individual GWA analysis results. Each HD GWA study (i.e., GWA1, 2, 3, 4, and 5) was independently analyzed in a linear mixed effect model to test the association of SNPs with sex and genetic ancestry covariates. Then, the 5 resultant sets were combined by meta-analysis using METAL (2011-03-25 release) (https://genome.sph.umich.edu/wiki/METAL_Documentation) (Willer et al., 2010). Finally, for the combined GWA12345 dataset, residual age at onset as the continuous dependent variable was modeled as a function of minor allele of the test SNP, sex, source of GWA, and first 4 principal component values from the genetic ancestry analysis in a linear mixed effect models with relationship matrix. Results of this combined continuous phenotype analysis served as the basis for the estimation of effect sizes and significances of SNPs.

#### Genome-wide association study (GWAS) analysis using the dichotomous phenotype

To confirm the lack of statistical artifacts in our standard combined continuous analysis and to reveal significant SNPs that are associated with our phenotype in a non-continuous manner, we additionally performed GWA analysis using a dichotomized phenotype. Subjects were sorted based on residual age at onset; those with the top 30% (2,719 subjects) and bottom 30% (2,719 subjects) were chosen and assigned to phenotype groups (‘late’ and ‘early’ onset, respectively). Based on the previous analysis of GWA123, a 30% cut-off provided the best ability to detect both the common and rare modifier effects at the chr 15 locus and so was used for this GWA12345 analysis. The dichotomous phenotype data were modeled as a function of minor allele count of test SNP, sex, source of GWA study, and first 4 principal components from the genetic ancestry analysis in a fixed effect model.

#### Conditional analysis

For selected candidate regions with significant association signals in the GWA12345 combined analysis using either continuous or dichotomous phenotype, we performed conditional analysis to characterize the number of independent modifier haplotypes. Having established a robust modifier effect at a locus, we then sought additional modifier haplotypes among the SNPs with suggestive signal in the overall analysis (p < 1E-5). Briefly, the same statistical model for single SNP analysis with an additional covariate of the minor allele count of the top SNP in the region was constructed in a fixed effect linear model to test independence of SNPs in the region. If significant association signals remained in the first conditional analysis, the top SNP in the conditional analysis was used for the next conditional analysis to confirm independence. Subsequently, SNPs tagging each independent modifier haplotype were identified based on continuous phenotype analysis results, dichotomous phenotype analysis results, conditional analysis, and eQTL data.

#### Association analysis using residual age at onset based on uninterrupted CAG repeat size

For samples tagged by the minor allele for rs764154313 or rs183415333 who were discovered to have a CAA-loss allele or a CAACAG-duplication allele on the chromosome with the expanded CAG repeat, we re-calculated residual age at onset based upon the true CAG repeat length. The true length for each of these HD subjects was then used as independent variable for our CAG-onset phenotyping model (Lee et al., 2012b). Then, re-calculated residual age at onset of motor symptoms was modeled as a function of the minor allele count of SNP, sex, source of GWA and genetic ancestry covariates in a mixed effect model. Dichotomous phenotype analysis (fixed effect model) was performed similarly. Overall association signals using residual age at onset based on genotyping CAG and those based on uninterrupted CAG repeat size were highly similar, except for the chromosome 4 region.

#### Modifier haplotypes and tagging SNPs at *MSH3/DHFR*

Comparison of continuous phenotype analysis and conditional analysis results revealed 3 modifier haplotypes at the locus at *MSH3/DHFR* on chromosome 5. The first haplotype, 5AM1, was defined by the top genome-wide significant SNP, rs701383, and further 5AM1 tag SNPs were those associated with hastened onset at p < 1E-5 in the continuous analysis and p > 1E-5 in analysis conditioned on rs701383 (red downward triangles in Figure 3). Haplotype 5AM2 was defined by rs113361582, which remained genome-wide significant in the analysis conditioned on rs701383. Further 5AM2 tag SNPs were those associated with delayed onset at p < 1E-3 in both the continuous analysis and analysis conditioned on rs701383 with minor allele frequency < 5% (green upward triangles in Figure 3). Haplotype 5AM3 was defined by rs1650742 which, at higher allele frequency than 5AM2 tag SNPs, was associated with an onset delaying signal (p < 2E-6) that was not reduced by conditioning on rs113361582. Further 5AM3 tag SNPs were those with minor allele frequency between 20 and 35% associated with delayed onset at p < 1E-3 in the continuous analysis, with p > 1E-3 in the conditional analysis using rs701383 (purple upward triangles in Figure 3).

#### Modifier haplotypes and tagging SNPs on chromosome 15

From association signals on chromosome 15, we identified 4 modifier haplotypes. Haplotype 15AM1 was marked by the top overall SNP, rs150393409, and additional 15AM1 tag SNPs were those with p < 5E-8 in the combined continuous phenotype analysis, p > 5E-8 in the conditional analysis using rs150393409, and p < 5E-8 in the conditional analysis using rs35811129, with minor allele frequency < 5% associated with hastened onset (red downward triangles in Figure 4). Haplotype 15AM2 was marked by rs35811129 and additional 15AM2 tag SNPs were those with p < 5E-8 in the combined continuous analysis, p < 5E-8 in the conditional analysis using rs150393409, p > 5E-8 in the conditional analysis using rs35811129, with minor allele frequency > 20% associated with delayed onset (green upward triangles in Figure 4). Haplotype 15AM3 was marked by rs151322829 which remained genome-wide significant in the above conditional analyses. Additional 15AM3 tag SNPs were those with p < 1E-5 in the combined continuous analysis and in conditional analyses with either rs150393409 or rs35811129, with minor allele frequency < 3% associated with hastened onset (purple downward triangles in Figure 4). Finally, haplotype 15AM4 was revealed by rs34017474, whose significance increased in the conditional analysis using rs150393409, and additional 15AM4 tag SNPs were those with p < 1E-5 in the continuous analysis, p < 5E-8 in the conditional analysis using rs150393409, and minor allele frequency > 30% (gold triangles in Figure 4).

#### Modifier haplotypes and tagging SNPs on chromosome 19

We identified 3 modifier haplotypes at the chromosome 19 locus. Haplotype 19AM1 was marked by the top genome-wide significant SNP, rs274883, and additional 19AM1 tag SNPs were those with p < 1E-4 in the combined continuous analysis, p > 1E-2 in the conditional analysis using rs274883 and associated with delayed onset (red upward triangles in Figure 5). Haplotype 19AM2 was marked by rs3730945 whose suggestive signal was not reduced by conditional analysis using rs274883. Additional 19AM2 tag SNPs were those with p < 1E-5 in the combined continuous analysis, p > 1E-5 in the conditional analysis using rs3730945, and minor allele frequency > 30%, associated with hastened onset (green downward triangles in Figure 5). Haplotype 19AM3 was revealed by rs145821638, whose suggestive signal was not reduced by conditional analysis using rs274883, and additional 19AM3 tag SNPs were those with p < 1E-2 in the combined continuous analysis, p > 1E-2 in the conditional analysis using rs145821638, and minor allele frequency < 2% associated with delayed onset (purple upward triangles in Figure 5).

#### Transcriptome-wide association study analysis

The association of gene expression and residual age at onset TWAS was performed using the FUSION package (Gusev et al., 2016), imputing the Common Mind Consortium prefrontal cortex expression to the GWA12345 summary association statistics, for the 5419 genes for which there was a significant genetic component of expression. For genes with a significant TWAS association (genome-wide significance p < 9.2E-6), the association between SNPs in the region and residual age at onset was calculated conditional on expression using FUSION. The proportion of the genetic liability to age at onset attributable to expression was quantified by using HESS to calculate regional heritability using the GWAS summary statistics before and after conditioning on expression (Shi et al., 2016). The ratio of the regional heritability after conditioning on expression to that before conditioning was regarded as an estimate of the proportion of genetic liability that is not attributable to expression (thus, 1 minus the ratio gives the proportion of liability that is attributable to expression).

#### Pathway analysis

Gene-wide association analyses were carried out in MAGMA (de Leeuw et al., 2015) on summary statistics from GWA12345, using genotypes from GWA345 as a reference panel to estimate LD. For primary analysis, a window of 35kb upstream and 10kb downstream of gene positions (GRCh37/hg19) was used with the “multi” analysis option, combining the mean SNP p value with the top SNP p value (corrected for number of SNPs, LD). Pathway enrichment analyses were performed in MAGMA, correcting for LD between genes, SNPs, initially using “self contained analysis” measuring overall association among genes in a pathway, and then using a more conservative “competitive” analysis, to compare association in genes within a pathway to those outside the pathway. ALIGATOR (Holmans et al., 2009) was used to test whether pathways contain a larger number of “significant” genes (here defined as the minimum SNP p value in that gene, chosen such that 5% of genes in the genome are considered significant), than expected by chance, given the number of SNPs they contain. Initial analysis considered the 14 pathways found to be significantly enriched for GWAS signal in GWA123 (Genetic Modifiers of Huntington’s Disease (GeM-HD) Consortium, 2015) augmented by 77 DNA repair pathways taken from Pearl et al. (Pearl et al., 2015). To test for potential novel areas of disease-relevant biology, an exploratory analysis was performed on 14,210 pathways containing between 10 and 500 genes from the Gene Ontology (GO) (Gene Ontology Consortium, 2015), Kyoto Encyclopedia of Genes and Genomes (KEGG) (Kanehisa et al., 2016), Mouse Genome Informatics (MGI) (Eppig et al., 2015), Pathway Interaction Database (PID) (Schaefer et al., 2009), Protein ANalysis THrough Evolutionary Relationships (PANTHER) (Mi et al., 2013), BioCarta (Nishimura, 2001) and Reactome (Fabregat et al., 2016).

#### Analysis of GTEx eQTL data

For selected candidate genes, we compared modification association signals obtained from either continuous phenotype analysis or dichotomous phenotype analysis to eQTL signals of human tissues obtained from GTEx consortium. Publicly available single tissue eQTL data (version 7) (https://gtexportal.org/home/) were downloaded. GTEx eQTL analysis calculated the effect size for a SNP relative to the alternative allele. In order to make comparable datasets, the sign of effect size of an eQTL SNP whose alternative allele is major allele in the HD dataset was flipped. Then, significance values (-log10(p value)) in HD modification GWA analysis were compared to those in GTEx eQTL analysis. SNPs from these comparisons were indicated based on the directions of eQTL; a SNP whose minor allele is associated with increased or decreased expression levels of test gene is indicated with an upward or downward triangle respectively.

#### Hardy Weinberg equilibrium test

The 7,013 GWA samples with peak proportional sum values in the CAG somatic expansion assay were rank sorted in descending order by peak proportional sum value and samples were divided into quartiles using Python v3.5.1., yielding 1753 samples in the quartile with the highest proportion of expanded PCR products. Chi square test for deviation of SNP rs701383 minor allele count from Hardy Weinberg equilibrium in this top quartile was performed according to the standard method using the formula: (Obs-Exp)^2^/Exp, where Obs = observed, Exp = expected. The expected minor allele count for each class was calculated from the minor allele frequency of the SNP in the entire 7,013 sample dataset. The minor allele frequency was 25.7% matching the minor allele frequency reported for Europeans.

#### Analysis Codes

1. Quality controls parameters of imputed SNPs were obtained based on the following options in the PLINK program.

plink–bfile GENOTYPE_DATA–freq

plink–bfile GENOTYPE_DATA–missing

plink–bfile GENOTYPE_DATA–hardy–nonfounders–hwe 0

Then, imputed SNPs were subjected to quality controls as described in the method section.

2. Construction of kinship matrix was based on the following code using genotype file in the PLINK format in the GEMMA program.

gemma -bfile GENOTYPE_DATA -gk 1

3. Association analysis using a linear mixed effect model was based on following code. GENOTYPE_DATA file set in the PLINK file format included the test phenotype in continuous phenotype.

gemma -bfile GENOTYPE_DATA -k KINSHIP_MATRIX -c COVARIATE -lmm 1 -maf 0

4. Meta-analysis was based on the following options in the METAL program.

MARKER SNP

ALLELE EFFECT_ALLELE OTHER_ALLELE

EFFECT SLOPE

PVALUE PVAL

WEIGHT N

PROCESS GWA1_RESULT

PROCESS GWA2_RESULT

PROCESS GWA3_RESULT

PROCESS GWA4_RESULT

PROCESS GWA5_RESULT

ANALYZE HETEROGENEITY

QUIT

5. GWA analysis of dichotomous phenotype was based on fixed effect model using following options. GENOTYPE_DATA file set in the PLINK file format included the test phenotype in dichotomous phenotype.

plink–bfile GENOTYPE_DATA–logistic–covar COVARIATE

6. QQ plots of GWA analysis were based on the R package ‘GenABEL’.

7. Conditional analyses of selected regions were based on the following options.

plink–bfile REGION_GENOTYPE_DATA–linear–covar COVARIATE–condition CONDITIONING_SNP

### Data and Code Availability

Original data will be made available on request. Data involving human subjects will be shared with qualified investigators given their institutional assurance that subject confidentiality will be ensured and that there will be no attempt to discover the identity of any human subject. For GWAS data, please direct inquiries to info@chdifoundation.org with the words ‘‘GWAS12345 data’’ in the subject line. The accession number for the GWAS summary statistics reported in this paper is DRYAD: https://datadryad.org/resource/doi:10.5061/dryad.5d4s2r8. Requests for further information or for other resources and reagents should be directed to James F. Gusella, Ph.D. (gusella@helix.mgh.harvard.edu).

## Consortia

The members of the Genetic Modifiers of Huntington’s Disease (GeM-HD) Consortium are: Group1: Jong-Min Lee, Kevin Correia, Jacob Loupe, Kyung-Hee Kim, Douglas Barker, Eun Pyo Hong, Michael J. Chao, Jeffrey D. Long, Diane Lucente, Jean Paul G. Vonsattel, Ricardo Mouro Pinto, Kawther Abu Elneel, Eliana Marisa Ramos, Jayalakshmi Srinidhi Mysore, Tammy Gillis, Vanessa C. Wheeler, Marcy E. MacDonald, and James F. Gusella; Group 2: Branduff McAllister^+^, Thomas Massey^+^, Christopher Medway, Timothy C. Stone, Lynsey Hall, Lesley Jones^&^, and Peter Holmans^&^; Group 3: Seung Kwak, Anka G. Ehrhardt, and Cristina Sampaio; Group 4: Marc Ciosi, Alastair Maxwell, Afroditi Chatzi, and Darren G. Monckton; Group 5: Michael Orth and G. Bernhard Landwehrmeyer on behalf of the European Huntington’s Disease Network (EHDN) Registry investigators; Jane S. Paulsen on behalf of the Huntington Study Group (HSG) PREDICT-HD investigators; E. Ray Dorsey and Ira Shoulson on behalf of the HSG COHORT, PHAROS and TREND-HD investigators; and Richard H. Myers on behalf of the HD-MAPS investigators. ^+^ and ^&^ indicate equal contributions.

## Supplemental Information

Document S1. Tables S1 and S3–S6

Table S2. Gene-Based Association, Related to Figure 1 and Table 1 Gene-wide association analyses using window of 35kb upstream and 10kb downstream of gene positions (as defined by NCBI Build 37) and no window are shown for GWA12345, GWA45, and GWA123, with genome-wide significant values, p < 2.5E-6 (Kiezun et al., 2012) shown in red, based upon the original CAG genotyping-based GWA12345 dataset.

Document S2. Consortium members with Affiliations
