# Supplementary material for: CAG Repeat Not Polyglutamine Length Determines Timing of Huntington’s Disease Onset
Source: Cell. 2019 Aug 8;178(4):887–900.e14. doi: 10.1016/j.cell.2019.06.036 (PMC6700281; doi:10.1016/j.cell.2019.06.036)
Supplement: Document S1. Tables S1 and S3–S6 [file mmc1.pdf]

Cell, Volume *178*

Supplemental Information

CAG Repeat Not Polyglutamine Length Determines Timing of Huntington's Disease Onset

Genetic Modifiers of Huntington's Disease (GeM-HD) Consortium

Supplementary Tables

Table S1 – *HTT* canonical and non-canonical DNA sequence variations in HD individuals and controls – related to Figure 2

The frequencies of DNA sequence variations immediately downstream to the uninterrupted *HTT* CAG repeat on expanded (>35 CAGs) and normal (<36 CAGs) chromosomes are given, classified as canonical (CAG repeat followed by a single CAACAG codon pair) and non-canonical (CAG repeat followed by all other sequence variations). A total of 1,980 sequences were determined by MiSeq DNA sequence analysis of 990 unique HD study individuals (17 without an expanded repeat) not having minor alleles for either of the chr 4 SNPs rs764154313 and rs183415333. Augmenting these results, a total of 9,486 sequences were observed in a large non-HD study sample of 4,738 unique individuals, as determined by analysis of whole genome sequence data. In the total of 11,456 chromosomes (986 with expanded CAG repeats and 10,470 with normal range CAG repeats), more than 95% of both expanded and control chromosomes had the canonical single penultimate CAA interruption, followed one of twenty-six different downstream sequence variations. The great majority of these canonical chromosomes matched the reference sequence (GRCh37/hg19): CAA1 CAG1 CCG1 CCA1 CCG7 CCT2 CAGCTTCCT1, or the common polymorphic variant: CAA1 CAG1 CCG1 CCA1 CCG10 CCT2 CAGCTTCCT1. The non-canonical sequences comprised twenty-one different sequence variations. A number of canonical and non-canonical sequence variations featured codon differences that alter the proline-rich amino acid sequence that follows the polyglutamine tract. On canonical chromosomes, two infrequent sequence variations exhibited a TCG serine or a ACG threonine residue amidst the proline codons. On four rare non-canonical sequence variations the CAG repeat was followed by a CAC histidine codon. One of these also featured a CGC arginine codon. Yet another non-canonical sequence displayed a CTA leucine codon and an ACG threonine codon. Neither frame-shift codons nor stop codons were observed in this large set of chromosomes.

Table S1 – *HTT* canonical and non-canonical DNA sequence variations in HD individuals and controls – related to Figure 2

Sequence adjacent to uninterrupted CAG repeat	HD Expanded (>35 CAG) chromosome (number)	HD Expanded (>35 CAG) chromosome (frequency)	HD Normal (<36 CAG) chromosome (number)	HD Normal (<36 CAG) chromosome (frequency)	Non-HD Expanded (>35 CAG) chromosome (number)	Non-HD Expanded (>35 CAG) chromosome (frequency)	Non-HD Normal (<36 CAG) chromosome (number)	Non-HD Normal (<36 CAG) chromosome (frequency)
**Canonical single CAA interruption**								
CAA1 CAG1 CCG1 CCA1 CCG7 CCT2 CAGCTTCCT1	789	81.09%	552	54.82%	12	92.31%	5,121	54.12%
CAA1 CAG1 CCG1 CCA1 CCG10 CCT2 CAGCTTCCT1	120	12.33%	311	30.88%	1	7.69%	3,055	32.28%
CAA1 CAG1 CCG9 CCT2 CAGCTTCCT1	6	0.62%	57	5.66%	0	0.00%	412	4.35%
CAA1 CAG1 CCG1 CCA1 CCG9 CCT2 CAGCTTCCT1	8	0.82%	23	2.28%	0	0.00%	407	4.30%
CAA1 CAG1 CCG1 CCA1 CCG9 CCT3 CAGCTTCCT1	1	0.10%	12	1.19%	0	0.00%	131	1.38%
CAA1 CAG1 CCG1 CCA1 CCG6 CCT2 CAGCTTCCT1	1	0.10%	5	0.50%	0	0.00%	12	0.13%
CAA1 CAG1 CCG10 CCT2 CAGCTTCCT1	0	0.00%	1	0.10%	0	0.00%	0	0.00%
CAA1 CAG1 CCG8 CCT2 CAGCTTCCT1	0	0.00%	1	0.10%	0	0.00%	0	0.00%
CAA1 CAG1 CCG1 CCA1 CCG5 CCT2 CAGCTTCCT1	1	0.10%	0	0.00%	0	0.00%	0	0.00%
CAA1 CAG1 CCG1 CCA1 CCG7 CCT3 CAGCTTCCT1	1	0.10%	0	0.00%	0	0.00%	0	0.00%
CAA1 CAG1 CCG1 CCA1 CCG1 CCA1 CCG10 CCT2 CAGCTTCCT1	0	0.00%	0	0.00%	0	0.00%	1	0.01%
CAA1 CAG1 CCG1 CCA1 CCG10 CCT3 CAGCTTCCT1	0	0.00%	0	0.00%	0	0.00%	1	0.01%
CAA1 CAG1 CCG1 CCA1 CCG11 CCT2 CAGCTTCCT1	0	0.00%	0	0.00%	0	0.00%	9	0.10%
CAA1 CAG1 CCG1 CCA1 CCG12 CCT2 CAGCTTCCT1	0	0.00%	0	0.00%	0	0.00%	8	0.08%
CAA1 CAG1 CCG1 CCA1 CCG3 ACG1 CCG3 CCT2 CAGCTTCCT1	0	0.00%	0	0.00%	0	0.00%	1	0.01%
CAA1 CAG1 CCG1 CCA1 CCG3 CAG1 CCG6 CCT2 CAGCTTCCT1	0	0.00%	0	0.00%	0	0.00%	1	0.01%
CAA1 CAG1 CCG1 CCA1 CCG4 CCT2 CAGCTTCCT1	0	0.00%	0	0.00%	0	0.00%	2	0.02%
CAA1 CAG1 CCG1 CCA1 CCG6 CCT3 CAGCTTCCT1	0	0.00%	0	0.00%	0	0.00%	3	0.03%
CAA1 CAG1 CCG1 CCA1 CCG8 CCT2 CAGCTTCCT1	0	0.00%	0	0.00%	0	0.00%	2	0.02%
CAA1 CAG1 CCG1 CCA1 CCG8 TCG1 CCG1 CCT2 CAGCTTCCT1	0	0.00%	0	0.00%	0	0.00%	1	0.01%
CAA1 CAG1 CCG1 CCC1 CCG9 CCT2 CAGCTTCCT1	0	0.00%	0	0.00%	0	0.00%	1	0.01%
CAA1 CAG1 CCG10 CCT1 CAGCTTCCT1	0	0.00%	0	0.00%	0	0.00%	1	0.01%
CAA1 CAG1 CCG11 CCT3 CAGCTTCCT1	0	0.00%	0	0.00%	0	0.00%	1	0.01%
CAA1 CAG1 CCG12 CCT2 CAGCTTCCT1	0	0.00%	0	0.00%	0	0.00%	4	0.04%
CAA1 CAG1 CCG4 CCT2 CAGCTTCCT1	0	0.00%	0	0.00%	0	0.00%	1	0.01%
CAA1 CAG1 CCT1 CCA1 CCG7 CCT2 CAGCTTCCT1	0	0.00%	0	0.00%	0	0.00%	1	0.01%
**Non-Canonical**								
CAA1 CAG1 CAA1 CAG1 CCG1 CCA1 CCG7 CCT3 CAGCTTCCT1	17	1.75%	42	4.17%	0	0.00%	261	2.76%
CCG1 CCA1 CCG7 CCT2 CAGCTTCCT1	8	0.82%	1	0.10%	0	0.00%	7	0.07%

CCG9 CCT2 CAGCTTCCT1	2	0.21%	1	0.10%	0	0.00%	0	0.00%
CCG1 CCA1 CCG10 CCT2 CAGCTTCCT1	0	0.00%	1	0.10%	0	0.00%	4	0.04%
CCG12 CCT2 CAGCTTCCT1	9	0.92%	0	0.00%	0	0.00%	2	0.02%
CCG10 CCT2 CAGCTTCCT1	5	0.51%	0	0.00%	0	0.00%	0	0.00%
CAA3 CAG1 CCG1 CCA1 CCG7 CCT2 CAGCTTCCT1	2	0.21%	0	0.00%	0	0.00%	0	0.00%
CAC1 CAG3 CAA1 CAG1 CAA1 CAG1 CCG1 CCA1 CCG7 CCT3 CAGCTTCCT1	2	0.21%	0	0.00%	0	0.00%	0	0.00%
CAC1 CAG3 CAA1 CAG1 CCG1 CCA1 CCG7 CCT2 CAGCTTCCT1	1	0.10%	0	0.00%	0	0.00%	0	0.00%
CAA1 CAG1 CAA1 CAG1 CCG1 CCA1 CCG7 CCC1 CCT2 CAGCTTCCT1	0	0.00%	0	0.00%	0	0.00%	1	0.01%
CAA1 CAG1 CAA1 CAG1 CCG1 CCA1 CCG7 CCT2 CAGCTTCCT1	0	0.00%	0	0.00%	0	0.00%	1	0.01%
CAA1 CCG10 CCT2 CAGCTTCCT1	0	0.00%	0	0.00%	0	0.00%	1	0.01%
CAA2 CAG1 CCG1 CCA1 CCG7 CCT2 CAGCTTCCT1	0	0.00%	0	0.00%	0	0.00%	1	0.01%
CAC1 CAG1 CCG1 CCC1 CCG10 CCT2 CAGCTTCCT1	0	0.00%	0	0.00%	0	0.00%	1	0.01%
CAC1 CGC1 CCG1 CCC1 CCG10 CCT2 CAGCTTCCT1	0	0.00%	0	0.00%	0	0.00%	1	0.01%
CCA1 CAG1 CCG1 CTA1 CCG4 ACG1 CCG5 CCT2 CAGCTTCCT1	0	0.00%	0	0.00%	0	0.00%	1	0.01%
CCG1 CAG2 CAA1 CAG1 CCG12 CCT2 CAGCTTCCT1	0	0.00%	0	0.00%	0	0.00%	1	0.01%
CCG13 CCT2 CAGCTTCCT1	0	0.00%	0	0.00%	0	0.00%	2	0.02%
CGG1 CAG1 CAA1 CAG1 CCG1 CCA1 CCG7 CCT2 CAGCTTCCT1	0	0.00%	0	0.00%	0	0.00%	1	0.01%
CGG1 CAG2 CAA1 CAG1 CCG1 CCA1 CCG7 CCT2 CAGCTTCCT1	0	0.00%	0	0.00%	0	0.00%	2	0.02%
**TOTAL**	**973**	**100.00%**	**1007**	**100.00%**	**13**	**100.00%**	**9,463**	**100.00%**

Table S3 –Enrichment in GWA12345 of pathways from GWA123 – related to Figure 1 and Table 1

Pathway enrichments relative to those reported in GWA123 (GeM-HD Consortium, 2015) are shown, for the original CAG genotyping-based GWA12345 dataset, from a “self contained analysis” measuring overall association among genes in a pathway, a “competitive” analysis measuring association in genes within a pathway to those outside the pathway and analysis with ALIGATOR to test whether pathways contain a larger number of “significant” genes than expected by chance, given the number of SNPs they contain.

Table S3 –Enrichment in GWA12345 of pathways from GWA123 – related to Figure 1 and Table 1

Pathway	#genes	self p(GWA12345)	p(GWA12345)	self p(GWA45)	p(GWA45)	p(ALIG-GWA12345)	p(ALIG-GWA45)	p(GWA123)	Description
GO: 32300	8	1.07E-26	4.77E-02	1.14E-09	2.67E-01	0.000550	0.008380	3.82E-05	mismatch repair complex
GO: 30983	12	1.57E-19	2.40E-02	3.04E-06	3.97E-01	0.002072	0.020160	7.43E-06	mismatched DNA binding
GO: 32389	4	1.14E-18	7.51E-02	9.38E-08	5.74E-01	0.000440	0.013200	1.66E-05	MutLalpha complex
KEGG 3430	22	7.87E-18	5.21E-03	1.76E-08	2.97E-02	0.000042	0.003260	6.65E-06	KEGG_MISMATCH_REPAIR
GO: 6298	28	3.22E-17	3.54E-03	4.52E-09	4.35E-02	0.000152	0.006980	3.25E-06	mismatch repair
GO: 4748	3	2.70E-09	7.20E-07	6.20E-04	1.01E-02	0.004220	NA	2.66E-05	ribonucleoside-diphosphate reductase activity
GO: 16728	3	2.70E-09	7.20E-07	6.20E-04	1.01E-02	0.004220	NA	2.66E-05	oxidoreductase activity
GO: 32407	5	2.54E-06	1.35E-01	9.91E-02	3.66E-01	0.011260	NA	5.74E-05	MutSalpha complex binding
GO: 33683	38	1.48E-01	3.54E-01	5.54E-03	1.08E-01	0.040420	0.147000	1.69E-06	nucleotide-excision repair
GO: 90200	29	1.49E-01	3.69E-01	1.74E-02	1.74E-01	NA	0.257000	8.89E-08	positive regulation of release of cytochrome c from mitochondria
GO: 10822	165	4.59E-01	6.88E-01	1.88E-02	3.93E-01	0.692200	0.255200	7.63E-05	positive regulation of mitochondrion organization
GO: 1900063	3	5.15E-01	9.28E-01	7.94E-02	4.69E-01	NA	NA	8.39E-05	regulation of peroxisome organization
GO: 90140	23	8.07E-01	6.36E-01	2.16E-01	1.59E-01	0.629500	NA	1.57E-05	regulation of mitochondrial fission
GO: 90141	15	8.85E-01	6.00E-01	5.34E-01	4.10E-01	NA	NA	2.30E-06	positive regulation of mitochondrial fission

Table S4 – Enrichment of pathways from a larger set of DNA repair pathways – related to Figure 1 and Table 1

Pathway enrichments are shown for a larger set of DNA repair pathways (Pearl et al., 2015).

Table S4 – Enrichment of pathways from a larger set of DNA repair pathways – related to Figure 1 and Table 1

Pathway	#genes	self p(GWA12345)	p(GWA12345)	self p(GWA45)	p(GWA45)	Description 1	Description 2	Description 3	Description 4
2071017	4	3.38E-26	3.66E-04	4.11E-08	4.99E-03	Repair_pathway	SSR	MMR	MutL_homologs
2071000	25	8.16E-23	5.53E-03	2.09E-10	3.19E-02	Repair_pathway	SSR	MMR	
2000000	219	1.44E-08	1.69E-02	1.07E-05	3.38E-02	Repair_pathway			
2071015	3	1.34E-07	1.89E-01	4.77E-04	2.69E-01	Repair_pathway	SSR	MMR	Mismatch_and_loop_recognition_factors
2070000	109	4.32E-07	9.93E-02	8.56E-04	3.93E-01	Repair_pathway	SSR		
2071020	4	6.74E-06	1.45E-01	2.05E-04	1.28E-01	Repair_pathway	SSR	MMR	Other_MMR_factors
1120000	6	1.46E-05	5.89E-03	3.32E-03	2.77E-02	Associated_process	Modulation_of_nucleotide_pools		
2111513	1	9.78E-05	2.76E-02	1.29E-04	3.01E-02	Repair_pathway	Associated_process	TLS	DNA_polymerases
2071104	9	2.23E-04	4.81E-02	2.61E-03	1.81E-01	Repair_pathway	SSR	BER	LONG_PATCH-BER_factors
1051200	6	1.54E-03	1.07E-01	4.00E-03	3.05E-02	Associated_process	Ubiquitin_response	Ubiquitin_ligases_(E3)	
2022100	4	3.09E-03	3.94E-01	3.83E-02	4.74E-01	Repair_pathway	DSR	Alt-NHEJ	
2020000	113	3.13E-03	5.30E-01	1.34E-03	1.33E-01	Repair_pathway	DSR		
1000000	160	1.04E-02	5.21E-02	7.60E-03	4.09E-02	Associated_process			
2070600	64	1.17E-02	6.39E-02	7.21E-02	1.94E-01	Repair_pathway	SSR	NER	
2110000	18	1.46E-02	5.60E-04	2.59E-01	6.14E-02	Repair_pathway	Associated_process		
2111500	18	1.46E-02	5.60E-04	2.59E-01	6.14E-02	Repair_pathway	Associated_process	TLS	
2070607	41	1.58E-02	8.50E-02	1.91E-02	9.53E-02	Repair_pathway	SSR	NER	TCR_(Transcription_coupled_repair)
1051221	1	2.11E-02	1.00E+00	4.76E-02	9.74E-01	Associated_process	Ubiquitin_response	Ubiquitin_ligases_(E3)	Other_single_Ring-_finger_type_E3
2071003	4	2.79E-02	4.22E-01	1.41E-02	8.05E-02	Repair_pathway	SSR	MMR	DNA_polymerase_delta
1010000	29	2.92E-02	2.55E-01	7.68E-02	4.59E-02	Associated_process	Chromatin_remodelling		
1083000	5	3.14E-02	7.38E-02	2.06E-02	7.51E-02	Associated_process	Checkpoint_factors	RAD9-Hus1-Rad1_complex	
1082200	2	4.22E-02	1.26E-01	4.81E-01	5.95E-01	Associated_process	Checkpoint_factors	damage_in_S_phase	
2071018	2	4.66E-02	2.64E-01	1.36E-01	8.07E-01	Repair_pathway	SSR	MMR	MutS_homologs_specialized_for_meiosis
1082900	1	5.52E-02	2.49E-01	2.73E-02	1.05E-01	Associated_process	Checkpoint_factors	Rad17-Mec3-_Ddc1_complex	
1080000	51	5.58E-02	3.40E-03	7.20E-02	2.08E-01	Associated_process	Checkpoint_factors		
2070603	10	6.87E-02	3.21E-01	1.21E-01	5.69E-01	Repair_pathway	SSR	NER	DNA_polymerase_delta
1050000	27	9.15E-02	4.57E-01	4.69E-02	3.77E-01	Associated_process	Ubiquitin_response		
2071023	3	1.02E-01	1.74E-01	2.00E-01	1.26E-01	Repair_pathway	SSR	MMR	RPA_(replication_factor_A)
2020200	50	1.08E-01	5.74E-01	3.41E-01	5.71E-01	Repair_pathway	DSR	HR_(Homologous_Recombination)	
2071100	41	1.08E-01	7.41E-01	7.48E-02	6.36E-01	Repair_pathway	SSR	BER	
2070605	4	1.17E-01	2.32E-01	5.39E-01	7.27E-01	Repair_pathway	SSR	NER	DNA_polymerase_epsilon
3000000	77	1.42E-01	9.68E-01	1.76E-01	8.75E-01	Genes_with_probable_DDR_role			
1082700	9	1.45E-01	1.54E-03	5.43E-01	3.19E-01	Associated_process	Checkpoint_factors	G2-M_checkpoint	
1082500	2	1.56E-01	4.67E-01	9.24E-02	8.64E-01	Associated_process	Checkpoint_factors	FPC_(fork_protection_complex)	
1080800	11	1.63E-01	2.78E-01	1.06E-01	5.18E-01	Associated_process	Checkpoint_factors	G1-CC_phase	
1051930	1	1.77E-01	2.31E-01	5.75E-02	4.81E-01	Associated_process	Ubiquitin_response	Ubiquitin-_conjugating_enzymes_(E2)	UBL-conjugating_enzymes
2020400	26	1.79E-01	6.18E-01	6.31E-02	2.53E-01	Repair_pathway	DSR	NHEJ	
2071010	5	1.99E-01	1.94E-01	4.13E-01	7.58E-01	Repair_pathway	SSR	MMR	RFC_(replication_factor_C)
1051209	1	2.25E-01	4.17E-01	8.87E-01	8.50E-01	Associated_process	Ubiquitin_response	Ubiquitin_ligases_(E3)	single_Ring-finger_type_E4
1051208	1	2.38E-01	1.50E-02	1.65E-01	2.02E-01	Associated_process	Ubiquitin_response	Ubiquitin_ligases_(E3)	single_Ring-finger_type_E3
1050500	9	2.86E-01	3.57E-01	4.50E-01	6.86E-01	Associated_process	Ubiquitin_response	Deubiquitinating_enzyme_(DUB)	
1050501	9	2.86E-01	3.57E-01	4.50E-01	6.86E-01	Associated_process	Ubiquitin_response	Deubiquitinating_enzyme_(DUB)	UBL-specific_proteases_(ULPs)
1081300	5	3.02E-01	3.54E-01	3.94E-01	7.47E-01	Associated_process	Checkpoint_factors	HRAD17(Rad24)-_RFC_complex	

1130000	2	3.09E-01	2.08E-02	9.01E-01	6.44E-01	Associated_process	Topisomerase_damage_reversal		
2072800	1	3.26E-01	5.65E-01	1.12E-01	4.10E-01	Repair_pathway	SSR	Other_SSR_genes	
2020300	12	3.33E-01	5.87E-01	4.42E-02	6.60E-01	Repair_pathway	DSR	Other_DSR_genes	
2071119	17	3.44E-01	7.53E-01	7.01E-01	8.56E-01	Repair_pathway	SSR	BER	Other_BER_factors
2021400	1	3.52E-01	1.63E-01	8.91E-01	5.85E-01	Repair_pathway	DSR	HR_(HomologousRecombination)	
1051222	1	3.61E-01	5.12E-02	7.85E-02	1.61E-01	Associated_process	Ubiquitin_response	Ubiquitin_ligases_(E3)	Riddle_syndrome!
2072300	3	3.74E-01	3.85E-01	6.54E-01	9.13E-01	Repair_pathway	SSR	Direct_Repair	
2020100	35	3.74E-01	5.65E-01	1.55E-01	3.19E-01	Repair_pathway	DSR	FA_(Fanconi_anemia_pathway)	
2111514	5	3.91E-01	4.93E-01	3.28E-01	2.75E-01	Repair_pathway	Associated_process	TLS	epistasis_group
1090000	9	3.99E-01	2.72E-01	3.56E-02	3.70E-01	Associated_process	p53_pathway		
2071124	3	4.44E-01	5.98E-01	6.26E-01	4.26E-01	Repair_pathway	SSR	BER	SHORT_PATCH-BER_factors
1080900	11	4.56E-01	2.20E-01	5.69E-01	9.29E-01	Associated_process	Checkpoint_factors	G1-S_checkpoint	
2111531	6	5.05E-01	6.52E-01	5.73E-01	1.58E-01	Repair_pathway	Associated_process	TLS	Y-family_DNA_polymerases
3060000	2	5.18E-01	7.12E-01	6.61E-01	5.07E-01	Genes_with_probable_DDR_role	Direct_Repair_(not_in_humans)		
1051700	4	5.49E-01	1.86E-01	4.77E-01	5.45E-01	Associated_process	Ubiquitin_response	Ubiquitin-like_proteins_(UBLs)	
1051725	4	5.49E-01	1.86E-01	4.77E-01	5.45E-01	Associated_process	Ubiquitin_response	Ubiquitin-like_proteins_(UBLs)	SUMO
1051900	4	5.54E-01	7.65E-01	2.50E-01	2.95E-01	Associated_process	Ubiquitin_response	Ubiquitin-_conjugating_enzymes_(E2)	
2070606	1	6.32E-01	6.76E-01	8.04E-01	3.68E-01	Repair_pathway	SSR	NER	DNA_polymerase_kappa
1031600	3	6.65E-01	3.22E-01	9.33E-01	5.49E-01	Associated_process	Telomere_maintenance	Alternative_mechanism	
1031616	3	6.65E-01	3.22E-01	9.33E-01	5.49E-01	Associated_process	Telomere_maintenance	Alternative_mechanism	MRN_Complex
1040000	14	6.75E-01	2.54E-01	6.29E-01	9.12E-01	Associated_process	Chromosome_segregation		
1052000	3	7.04E-01	7.79E-01	4.11E-01	7.67E-01	Associated_process	Ubiquitin_response	Ubiquitins_and_Ubiquitin-like_proteins	
1052028	3	7.04E-01	7.79E-01	4.11E-01	7.67E-01	Associated_process	Ubiquitin_response	Ubiquitins_and_Ubiquitin-like_proteins	Ubiquitins
1051800	1	7.33E-01	9.66E-01	5.20E-01	8.08E-01	Associated_process	Ubiquitin_response	Ubiquitin-_activating_enzymes_(E1)	
1051829	1	7.33E-01	9.66E-01	5.20E-01	8.08E-01	Associated_process	Ubiquitin_response	Ubiquitin-_activating_enzymes_(E1)	UBL-activating_enzymes
2072400	1	7.36E-01	9.46E-01	6.23E-01	8.75E-01	Repair_pathway	SSR	DNA_replication	
1051927	3	7.56E-01	8.70E-01	5.52E-01	2.86E-01	Associated_process	Ubiquitin_response	Ubiquitin-_conjugating_enzymes_(E2)	Ubiquitin-conjugating_enzymes
1030000	26	7.77E-01	9.95E-01	4.47E-01	2.46E-01	Associated_process	Telomere_maintenance		
1080700	1	8.04E-01	8.71E-01	4.69E-01	5.07E-01	Associated_process	Checkpoint_factors	S-CC_phase	
2070602	6	8.20E-01	4.18E-01	8.35E-01	4.94E-01	Repair_pathway	SSR	NER	GGR_(Global_genome_repair)
2071112	11	8.24E-01	9.62E-01	1.64E-01	6.44E-01	Repair_pathway	SSR	BER	DNA_glycosylases
1082600	2	8.30E-01	9.05E-01	6.13E-01	2.43E-01	Associated_process	Checkpoint_factors	G2-CC_phase	
2071111	1	9.02E-01	8.26E-01	3.95E-01	1.75E-01	Repair_pathway	SSR	BER	AP_endonucleases
1100000	3	9.68E-01	9.36E-01	6.34E-01	9.65E-02	Associated_process	DNA_replication		

Table S5 – Top scoring genes in pathways from Tables S3 and S4 – related to Figure 1 and Table 1

Entrez	Gene Symbol	Chr	Start	End	#SNPs	p(GWA12345)	p(GWA45)	#SNPs(GWA123)	p(GWA123)	Pathways	Pearl Pathways
22909	*FAN1*	15	31191703	31235311	287	1.75E-35	1.11E-17	114	1.68E-09	GO:33683	2000000
3978	*LIG1*	19	48618702	48673852	455	3.77E-11	8.04E-11	258	6.39E-02	GO:6298, KEGG:3430	2000000, 2070000, 2071000, 2071020
4292	*MLH1*	3	37034841	37092337	301	1.01E-10	6.51E-03	128	3.91E-04	GO:6298, GO:30983, GO:32300, GO:32389, GO:32407, KEGG:3430	2000000, 2070000, 2071000, 2071017
50484	*RRM2B*	8	1.03E+08	1.03E+08	336	1.19E-10	9.20E-04	128	5.91E-06	GO:4748, GO:16728	NA
4437	*MSH3*	5	79950467	80172634	1126	1.20E-10	9.05E-07	781	1.98E-02	GO:6298, GO:30983, GO:32300, KEGG:3430	2000000, 2070000, 2071000, 2071015
4E+05	*POLN*	4	2073645	2230958	711	4.06E-10	1.29E-01	400	3.58E-02	NA	2000000
5395	*PMS2*	7	6012870	6048737	355	2.62E-09	7.33E-05	131	1.76E-05	GO:6298, GO:30983, GO:32300, GO:32389, GO:32407, KEGG:3430	2000000, 2070000, 2071000, 2071017
3064	*HTT*	4	3076408	3245687	796	5.31E-09	9.25E-06	372	4.63E-01	GO:10822	NA
5378	*PMS1*	2	1.91E+08	1.91E+08	370	9.09E-08	1.11E-04	214	7.08E-02	GO:6298, GO:30983, GO:32300, GO:32389	2000000, 2070000, 2071000, 2071017
10721	*POLQ*	3	1.21E+08	1.21E+08	475	9.78E-05	1.29E-04	263	7.15E-03	NA	2000000
27030	*MLH3*	14	75480467	75518235	206	1.42E-04	2.26E-01	63	6.39E-03	GO:6298, GO:30983, GO:32300, KEGG:3430	2000000, 2070000, 2071000, 2071017
6240	*RRM1*	11	4115791	4160116	427	4.41E-04	2.94E-02	133	3.37E-01	GO:4748, GO:16728	NA
5424	*POLD1*	19	50887531	50921275	283	5.03E-04	7.96E-02	85	2.09E-01	GO:6298, GO:33683, KEGG:3430	2000000, 2070000, 2071000
56852	*RAD18*	3	8918880	9005159	634	8.03E-04	1.79E-02	362	3.05E-01	NA	2000000
27113	*BBC3*	19	47724079	47736023	123	2.83E-03	1.30E-01	9	2.75E-01	GO:10822, GO:90200	NA
2956	*MSH6*	2	48010221	48034092	268	7.70E-03	4.25E-01	95	9.18E-02	GO:6298, GO:30983, GO:32300, KEGG:3430	2000000, 2070000, 2071000, 2071015

Table S6 – Enrichment across 14,210 pathways – related to Figure 1

Significant enrichments (self-contained analysis) in 14,210 pathways containing between 10 and 500 genes from the Gene Ontology (GO), Kyoto Encyclopedia of Genes and Genomes (KEGG), Mouse Genome Informatics (MGI), National Cancer Institute (NCI), Protein ANalysis THrough Evolutionary Relationships (PANTHER), BioCarta and Reactome.

Table S6 – Enrichment across 14,210 pathways – related to Figure 1

Pathway	#genes	self p(GWA12345)	p(GWA12345)	self p(GWA45)	p(GWA45)	p(ALIG-GWA12345)	p(ALIG-GWA45)	p(GWA123)	Description
GO: 32300	8	1.07E-26	4.77E-02	1.14E-09	2.67E-01	0.000550	0.008380	3.82E-05	mismatch repair complex
REACTOME 5358508	15	1.36E-19	1.70E-02	4.51E-10	9.02E-03	0.000054	0.006200	NA	Mismatch_Repair
GO: 30983	12	1.57E-19	2.40E-02	3.04E-06	3.97E-01	0.002072	0.020160	7.43E-06	mismatched DNA binding
REACTOME 5358606	14	2.56E-18	4.25E-03	1.66E-10	1.68E-03	0.000050	0.005520	NA	Mismatch_repair_(MMR)_directed_by_MSH2:MSH3_(MutSbeta)
KEGG 3430	22	7.87E-18	5.21E-03	1.76E-08	2.97E-02	0.000042	0.003260	6.65E-06	KEGG_MISMATCH_REPAIR
GO: 6298	28	3.22E-17	3.54E-03	4.52E-09	4.35E-02	0.000152	0.006980	3.25E-06	mismatch repair
REACTOME 5358565	14	1.51E-14	9.85E-02	2.09E-07	3.42E-02	0.000218	0.025100	NA	Mismatch_repair_(MMR)_directed_by_MSH2:MSH6_(MutSalpha)
GO: 16446	11	2.69E-13	1.41E-01	7.63E-05	2.13E-01	0.007250	0.066900	1.31E-03	somatic hypermutation of immunoglobulin genes
GO: 2566	12	2.19E-11	1.58E-01	2.30E-04	2.32E-01	0.008858	0.075720	1.31E-03	somatic diversification of immune receptors via somatic mutation
GO: 6281	466	5.35E-11	1.62E-02	3.64E-08	2.80E-02	0.170100	0.076940	1.21E-02	DNA repair
GO: 16445	26	3.16E-10	5.72E-02	8.94E-06	9.18E-02	0.003672	0.021840	7.82E-02	somatic diversification of immunoglobulins
GO: 2200	37	4.64E-10	4.57E-02	4.19E-05	6.85E-02	0.000126	0.004800	7.67E-02	somatic diversification of immune receptors
GO: 1990391	33	9.81E-10	1.40E-01	3.46E-04	4.36E-01	0.000012	0.016420	NA	DNA repair complex
GO: 51224	190	3.46E-09	1.03E-03	2.80E-05	2.25E-01	0.166700	0.387000	6.09E-01	negative regulation of protein transport
GO: 1904950	194	3.63E-09	8.05E-04	5.85E-05	2.29E-01	0.111900	0.415900	NA	negative regulation of establishment of protein localization
GO: 2377	41	5.12E-09	1.98E-02	4.49E-06	7.52E-02	0.010700	0.010340	1.32E-01	immunoglobulin production
GO: 98794	398	5.30E-09	5.34E-03	1.45E-07	1.02E-01	0.433600	0.353200	NA	postsynapse
GO: 5769	313	7.81E-09	3.87E-01	4.18E-10	3.78E-01	0.405100	0.107900	4.98E-01	early endosome
GO: 2562	31	7.34E-08	2.92E-02	4.86E-04	4.82E-02	0.001558	0.042260	9.34E-02	somatic diversification of immune receptors via germline recombination within a single locus
GO: 16444	31	7.34E-08	2.92E-02	4.86E-04	4.82E-02	0.001558	0.042260	9.34E-02	somatic cell DNA recombination
MGI: 2144	230	7.93E-08	2.59E-02	1.11E-06	2.42E-01	0.062430	0.008880	5.85E-01	abnormal_B_cell_differentiation
GO: 4175	386	1.26E-07	1.32E-01	6.12E-05	7.18E-01	0.014100	0.734100	8.32E-02	endopeptidase activity
GO: 34138	10	1.39E-07	1.08E-03	6.79E-07	1.03E-03	0.017340	0.117300	4.38E-01	toll-like receptor 3 signaling pathway
GO: 30425	469	1.49E-07	4.84E-01	6.26E-07	4.91E-01	0.476000	0.325700	4.52E-01	dendrite
GO: 98609	420	1.66E-07	9.90E-02	5.83E-06	7.94E-01	0.318800	0.138100	NA	cell-cell adhesion
GO: 97447	470	1.79E-07	5.00E-01	6.98E-07	5.02E-01	0.491600	0.339600	NA	dendritic tree
GO: 42578	344	1.95E-07	3.69E-02	1.23E-02	7.13E-01	0.671400	0.753100	4.12E-01	phosphoric ester hydrolase activity
GO: 21537	227	2.08E-07	7.95E-04	1.72E-02	6.33E-01	0.035690	0.643700	2.94E-01	telencephalon development
GO: 2440	63	2.18E-07	4.89E-03	9.27E-05	2.23E-01	0.023730	0.067460	9.57E-02	production of molecular mediator of immune response
GO: 98742	214	2.36E-07	1.60E-01	1.79E-06	5.38E-01	0.253100	0.011140	NA	cell-cell adhesion via plasma-membrane adhesion molecules
GO: 51962	430	2.38E-07	7.13E-02	1.80E-10	6.95E-02	0.490900	0.332100	2.17E-01	positive regulation of nervous system development
GO: 30900	349	2.41E-07	1.10E-02	7.41E-04	1.74E-01	0.109300	0.726000	3.17E-01	forebrain development
GO: 44440	459	2.43E-07	1.74E-01	4.64E-05	7.17E-01	0.230100	0.166300	6.38E-01	endosomal part
GO: 2460	116	2.88E-07	1.76E-03	6.78E-03	5.65E-01	0.104700	0.204100	5.28E-01	adaptive immune response based on somatic recombination of immune receptors built from immunoglobulin superfamily domains
MGI: 11087	459	3.14E-07	6.47E-02	5.34E-07	8.30E-02	0.404600	0.486700	NA	neonatal_lethality_complete_penetrance
GO: 51051	440	3.18E-07	1.99E-03	4.05E-06	1.48E-01	0.439500	0.434400	1.21E-01	negative regulation of transport

MGI:	5460	466	4.20E-07	1.71E-02	1.31E-05	8.55E-01	0.142000	0.066040	NA	abnormal_leukopoiesis
MGI:	11085	375	4.50E-07	3.11E-01	2.58E-04	8.89E-01	0.428800	0.883500	NA	postnatal_lethality_complete_penetrance
GO:	16447	21	4.62E-07	3.82E-02	2.58E-04	6.91E-02	0.052110	0.215500	8.96E-02	somatic recombination of immunoglobulin gene segments
GO:	45910	20	5.01E-07	2.07E-01	6.11E-07	9.16E-02	0.019630	0.087820	8.57E-03	negative regulation of DNA recombination
GO:	45725	15	5.16E-07	6.74E-05	4.76E-05	9.74E-03	0.036960	NA	8.64E-01	positive regulation of glycogen biosynthetic process
GO:	16887	414	5.54E-07	4.86E-01	1.90E-02	8.94E-01	0.141500	0.700200	4.78E-01	ATPase activity
GO:	3682	467	5.56E-07	6.46E-01	1.87E-06	5.55E-01	0.555100	0.016240	9.18E-01	chromatin binding
GO:	19724	62	5.92E-07	1.41E-03	1.84E-02	5.02E-01	0.164500	0.367900	3.73E-01	B cell mediated immunity
GO:	5979	25	6.60E-07	8.74E-04	9.98E-04	8.58E-02	0.029410	NA	6.24E-01	regulation of glycogen biosynthetic process
GO:	10962	25	6.60E-07	8.74E-04	9.98E-04	8.58E-02	0.029410	NA	6.24E-01	regulation of glucan biosynthetic process
GO:	1654	325	6.81E-07	2.11E-01	4.27E-04	4.73E-01	0.303300	0.689000	8.60E-01	eye development
GO:	35239	325	6.85E-07	7.93E-02	1.13E-07	4.75E-02	0.553000	0.084960	7.67E-01	tube morphogenesis
MGI:	4816	62	6.98E-07	1.56E-02	7.32E-08	1.36E-01	0.006666	0.022480	6.22E-01	abnormal_class_switch_recombination
REACTOME	73894	300	7.03E-07	3.93E-02	3.06E-06	1.71E-01	0.024920	0.006340	NA	DNA_Repair
MGI:	2367	63	7.73E-07	2.55E-05	5.09E-04	9.65E-02	0.021080	0.056040	2.36E-01	abnormal_thymus_lobule_morphology
GO:	70875	16	7.89E-07	4.33E-05	1.29E-04	7.82E-03	0.038380	NA	8.31E-01	positive regulation of glycogen metabolic process
GO:	7005	394	1.14E-06	9.59E-04	6.55E-04	3.12E-01	0.581400	0.475000	2.73E-01	mitochondrion organization
GO:	46649	327	1.15E-06	2.64E-02	5.18E-03	9.21E-01	0.274200	0.371100	5.50E-01	lymphocyte activation
MGI:	2401	398	1.25E-06	5.60E-02	8.63E-05	9.20E-01	0.105700	0.069180	NA	abnormal_lymphopoiesis
GO:	3002	301	1.35E-06	5.80E-02	2.20E-03	7.95E-02	0.579400	0.574700	4.66E-01	regionalization
GO:	45296	296	1.35E-06	8.78E-01	5.33E-05	1.66E-01	0.265700	0.266100	5.99E-01	cadherin binding
GO:	50709	109	1.52E-06	1.66E-04	5.03E-06	1.21E-01	0.147500	0.145400	6.20E-01	negative regulation of protein secretion
GO:	1673	15	1.71E-06	3.57E-02	1.91E-01	3.45E-01	0.130400	0.129400	2.02E-02	male germ cell nucleus
GO:	2250	257	1.72E-06	1.91E-02	2.16E-02	9.04E-01	0.063150	0.530500	5.91E-01	adaptive immune response
GO:	9165	180	1.92E-06	1.94E-03	4.54E-05	2.75E-02	0.383800	0.046440	5.44E-01	nucleotide biosynthetic process
GO:	2000241	132	1.99E-06	6.53E-02	2.50E-04	3.84E-03	0.530100	0.118100	NA	regulation of reproductive process
GO:	51054	206	2.05E-06	4.40E-02	4.03E-04	8.75E-01	0.366300	0.252500	9.86E-01	positive regulation of DNA metabolic process
GO:	43010	282	2.20E-06	3.04E-01	8.72E-04	4.67E-01	0.247200	0.545900	8.58E-01	camera-type eye development
GO:	10720	435	2.22E-06	1.29E-01	3.02E-08	2.89E-01	0.727800	0.486200	4.48E-01	positive regulation of cell development
GO:	16064	61	2.28E-06	1.46E-03	3.75E-02	5.72E-01	0.160900	0.362300	3.53E-01	immunoglobulin mediated immune response
GO:	2449	110	2.30E-06	9.83E-04	3.27E-02	7.73E-01	0.045830	0.385500	3.01E-01	lymphocyte mediated immunity
MGI:	1263	246	2.36E-06	1.34E-01	1.02E-03	8.79E-01	0.237900	0.762100	NA	weight_loss
MGI:	1698	473	2.41E-06	6.44E-01	8.72E-08	5.38E-05	0.197400	0.029940	1.66E-01	decreased_embryo_size
GO:	32947	71	2.44E-06	1.32E-02	2.98E-04	2.28E-01	0.004226	0.162400	6.09E-01	protein complex scaffold activity
GO:	51052	363	2.54E-06	1.64E-01	6.23E-05	8.67E-01	0.773700	0.061080	2.22E-01	regulation of DNA metabolic process
MGI:	8932	225	2.73E-06	6.93E-01	6.10E-03	6.59E-01	0.117800	0.526300	3.01E-01	abnormal_embryonic_tissue_physiology
GO:	43254	371	2.77E-06	6.33E-01	4.52E-05	3.77E-02	0.117300	0.416000	2.03E-01	regulation of protein complex assembly
GO:	2792	115	2.78E-06	2.60E-04	2.99E-05	2.23E-01	0.108800	0.205400	7.55E-03	negative regulation of peptide secretion
MGI:	691	348	2.79E-06	6.24E-01	1.86E-03	8.34E-01	0.691900	0.285300	7.39E-01	enlarged_spleen
GO:	45830	22	2.88E-06	1.45E-02	2.06E-02	2.48E-01	0.004626	NA	6.46E-01	positive regulation of isotype switching
GO:	1901293	183	2.96E-06	3.17E-03	5.51E-05	4.77E-02	0.405500	0.052940	5.64E-01	nucleoside phosphate biosynthetic process
