# Supplementary material for: CAG Repeat Not Polyglutamine Length Determines Timing of Huntington’s Disease Onset
Source: Cell. 2019 Aug 8;178(4):887–900.e14. doi: 10.1016/j.cell.2019.06.036 (PMC6700281; doi:10.1016/j.cell.2019.06.036)
Supplement: Document S2. Consortium members with Affiliations [file mmc3.pdf]

Genetic Modifiers of Huntington's Disease (GeM-HD) Consortium

Jong-Min Lee[1,2#], Kevin Correia[1], Jacob Loupe[1,2], Kyung-Hee Kim[1,2], Douglas Barker[1], Eun Pyo Hong[1,2], Michael J. Chao[1,2], Jeffrey D. Long[3], Diane Lucente[1,2], Jean Paul G. Vonsattel[4], Ricardo Mouro Pinto[1,2], Kawther Abu Elneel[1], Eliana Marisa Ramos[1], Jayalakshmi Srinidhi Mysore[1], Tammy Gillis[1], Vanessa C. Wheeler[1,2,#], Marcy E. MacDonald[1,2,6,#] and James F. Gusella[1,4,5,6*,#,%]

[1]Molecular Neurogenetics Unit, Center for Genomic Medicine, Massachusetts General Hospital, Boston MA 02114, USA

[2]Department of Neurology, Harvard Medical School, Boston MA 02115, USA

[3]Department of Biostatistics, College of Public Health, and Department of Psychiatry, Carver College of Medicine, University of Iowa, Iowa City 52242, USA

[4]Department of Pathology and Cell Biology and the Taub Institute for Research on Alzheimer's Disease and the Aging Brain, Columbia University Medical Center, New York, NY 10032, USA

[5]Department of Genetics, Blavatnik Institute, Harvard Medical School, Boston MA 02115, USA

[6]Medical and Population Genetics Program, the Broad Institute of M.I.T. and Harvard, Cambridge MA 02142, USA

Branduff McAllister[7,+], Thomas Massey[7+], Christopher Medway[7], Timothy C. Stone[7], Lynsey Hall[7], Lesley Jones[7#&], Peter Holmans[7#&]

[7]Medical Research Council (MRC) Centre for Neuropsychiatric Genetics and Genomics, Division of Psychological Medicine and Clinical Neurology, School of Medicine, Cardiff University, Cardiff, United Kingdom

Seung Kwak[8#], Anka G. Ehrhardt[8] and Cristina Sampaio[8]

[8]CHDI Management/CHDI Foundation, Princeton NJ 08540 USA

Marc Ciosi[9], Alastair Maxwell[9], Afroditi Chatzi[9] and Darren G. Monckton[9]

[9]Institute of Molecular, Cell and Systems Biology, College of Medical, Veterinary and Life Sciences, University of Glasgow, Glasgow G12 8QQ, U.K

Michael Orth[10,#] and G. Bernhard Landwehrmeyer[10] on behalf of the European Huntington's Disease Network (EHDN) Registry investigators[^], Jane S. Paulsen[11] on behalf of the Huntington Study Group (HSG) PREDICT-HD investigators[^], E. Ray Dorsey[12] and Ira Shoulson[12] on behalf of the HSG COHORT, PHAROS and TREND-HD investigators[^], and Richard H. Myers[13,#] on behalf of the HD-MAPS investigators^

[10]Department of Neurology, University of Ulm, Germany

[11]Departments of Psychiatry and Neurology, University of Iowa Roy and Lucille Carver College of Medicine, Iowa City IA 52242, USA

[12]Department of Neurology, University of Rochester Medical Center, Rochester NY 14642, USA

[13]Department of Neurology and Genome Science Institute, Boston University School of Medicine, Boston MA 02118, USA

*Correspondence: James F. Gusella,

Footnotes:

[%] Denotes Lead Contact

[#] Denotes founding GeM-HD investigators

[+] equal contributions

[&] equal contributions

[^] The Acknowledgments section indicates links to the Enroll-HD, Registry, PREDICT-HD, COHORT, PHAROS, TREND-HD and HD-MAPS investigators
